# Neuroprotective effects of total phenolics from *Hemerocallis citrina* Baroni leaves through the PI3K/AKT pathway

**DOI:** 10.3389/fphar.2024.1370619

**Published:** 2024-07-12

**Authors:** Yanjun Jia, Yanping Wang, Zixia Wang, Zeyu Zhang, Ju Zhang, Jingjing Zhang, Ke Sun, Yongchen Hua, Guolin Chai, Fangdi Hu

**Affiliations:** ^1^ School of Pharmacy, Lanzhou University, Lanzhou, China; ^2^ Lanzhou Foci Pharmaceutical Co. Ltd., Lanzhou, China

**Keywords:** *Hemerocallis citrina* Baroni leaves, total phenolics, network pharmacology, PI3K/AKT, neuroprotection

## Abstract

Neurological injury, as a major pathogenic mechanism in depression, holds significant importance in the research and development of antidepressant drugs. *Hemerocallis citrina* Baroni (*H. citrina*), referred to as “Forgetting Sadness Grass,” has been confirmed to possess remarkable neuroprotective effects. Studies have identified that the total phenolics in *H. citrina* Baroni leaves (HLTP) consist of flavonoids and phenolic acids and numerous studies have substantiated the neuroprotective effects of them. Based on this, we propose that HLTP may possess neuroprotective properties. To confirm this hypothesis, we initially employed network pharmacology techniques to predict potential targets for the neuroprotective effects of HLTP based on the Swiss Target Prediction database. GO and KEGG analyses were conducted to predict potential pathways, and a component-target-pathway network was constructed. Molecular docking experiments were then performed to analyze the binding abilities of the selected active components with the main targets. Furthermore, we validated the neuroprotective effects of HLTP and key targets selected through network pharmacology using a corticosterone-induced PC12 neuronal cell damage model. Network pharmacology research has identified that in the HLTP, Quercetin, Rutin, Apigenin, and Isoquercitrin are potential active components that may exert neuroprotective effects by modulating key targets such as AKT1, TNF, TP53, and CASP3 through crucial pathways including PI3K/AKT and apoptosis. Molecular docking revealed that 4-O-Caffeoylquinic acid, 5-O-Caffeoylshikimic acid, 4-p-Coumaroylquinic acid, and 5-O-Feruloylquinic acid exhibit low binding energies with key targets. Particularly, 4-O-Caffeoylquinic acid forms stable binding through hydrogen bonding with residues such as LYS389, GLU49, GLN47, LYS30, ASP44, and GLU40 in AKT1. PC12 cells were stimulated with 200 μmol/L Corticosterone (Cort) for 24 h, and then treated with 50, 100 and 200 μg/mL of HLTP for 24 h. The cell viability of damaged cells were significantly increased in a dose-dependent manner by 9.50%, 10.42% and 21.25%, respectively (*P* < 0.01). Western blot analysis confirmed that HLTP significantly (*P* < 0.01) increased the protein expression of PI3K and AKT by 15.24%, 30.44%, 41.03%, and 21.78%, 43.63%, 12.86%, respectively. In addition, through biochemical method, flow cytometry and WB analysis, we found that different concentrations of HLTP can all improve cell damage by reducing ROS, MDA, Ca^2+^, Cyt-C, Caspase-3, TNF-α and IL-1β, and increasing SOD, CAT, MMP, Bcl-2/Bax and IL-10. In particular, the HLTP at 200 μg/mL, compared with the Model group, decreased by 140.2%, 54.66%, 51.34%, 65.26%, 40.32%, 63.87%, and 55.38%, and increased by 39.65%, 35.45%, 38.38%, 28.54%, and 39.98%, respectively. Through the above experiments, we verified that HLTP may exert neuroprotective effects by mediating the PI3K/AKT signaling pathway to counteract oxidative stress damage, improve mitochondrial dysfunction, and alleviate inflammatory injury.

## Graphical Abstract

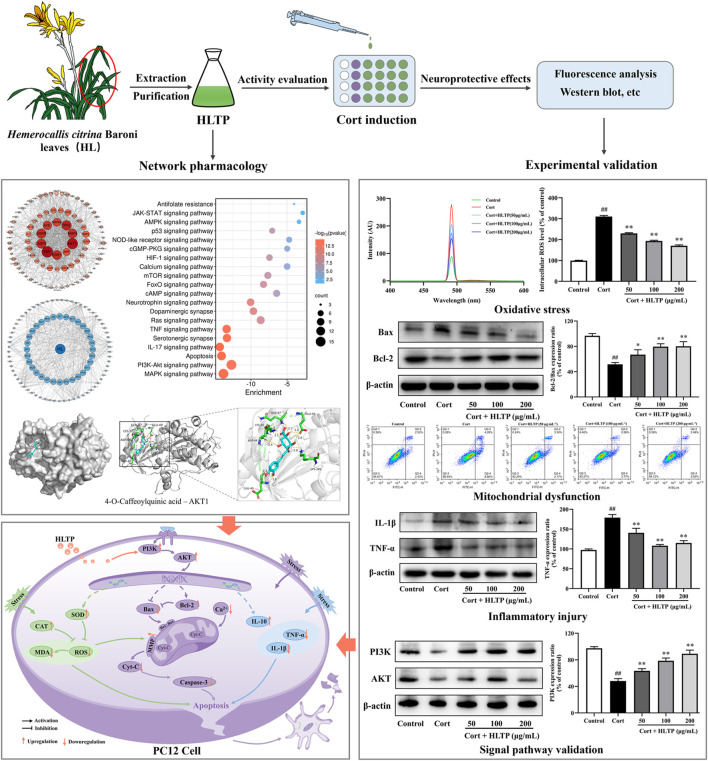

## 1 Introduction

Depression is a common mood disorder with a high prevalence and mortality rate. Approximately 350 million people globally suffer from depression, making it the third-largest disease burden worldwide (Xiong et al., 2020; Herzog et al., 2021). The etiology of depression is complex, resulting from the combined effects of various factors, including social environment, economic status, life events, and stress (Lee and Jung, 2024). The stress hypothesis mainly involves the activation of the Hypothalamic-Pituitary-Adrenal (HPA) axis, leading to the synthesis of Glucocorticoids (GCs) by the adrenal cortex. The secretion and levels of GCs can be regulated through the negative feedback inhibition of the HPA axis (Santen et al., 2018). However, under extreme or chronic stress, elevated concentrations of GCs can disrupt the functioning of the HPA axis negative feedback mechanism, resulting in excessive GCs secretion and damage to the nervous system, leading to psychiatric disorders such as depression (Dinkel et al., 2003). The stressors of depression can be physiological or psychologicaltressors. Numerous studies indicate that long-term stress-induced inflammation, oxidative stress, and mitochondrial dysfunction can induce central nervous system neuronal damage, which is a significant mechanism underlying the onset of depression (de Kloet et al., 2005; Cohen et al., 2007). Antidepressant drugs are generally believed to exert their antidepressant effects through neuroprotection. Consequently, improving neuronal damage induced by external stress has become a potential target for treating depression.

At present, the treatment of depression mainly relies on chemical drugs, but due to their inevitable side effects, finding safe, effective, and low side effect antidepressants is still a hot topic in pharmaceutical research (Xie et al., 2024). Traditional Chinese medicine (TCM), which is effective, low in toxicity, and cost-effective, has garnered considerable attention from researchers (Chen et al., 2022). Phenolic components in TCM have been proven to possess significant neuroprotective activity (Dalmagro et al., 2017). *Hemerocallis citrina* Baroni, commonly known as daylily, is a perennial herbaceous plant in the lily family (Liang et al., 2023). It has been used as both food and traditional medicine in East Asia for thousands of years. The dried flowers of *H. citrina* are typically consumed as a food item (Hao et al., 2022). Modern pharmacology has demonstrated that *H. citrina* has pharmacological effects, including immune regulation, antioxidation, and lipid-lowering (Li et al., 2017). In recent years, multiple studies have shown that *H. citrina* flowers exhibit significant neuroprotective activity, and the phenolic components in these flower buds contribute to this neuroprotective effect (Liu et al., 2022; Tian et al., 2017). The leaves of *H. citrina* are often discarded, leading to significant resource wastage. However, through systematic identification of the HLTP, we have discovered that they also contain abundant phenolic components, including flavonoids and phenolic acids. It remains unclear whether the collective action of these phenolic components in *H. citrina* leaves possesses neuroprotective effects and what the underlying mechanisms might be.

Network pharmacology, based on the structural and functional similarities between drugs, utilizes systems biology techniques and database resources to analyze target molecules, and analyze the molecular correlation laws between drugs and treatment targets (Zhang et al., 2023). Network pharmacology has been widely applied in the study of the therapeutic mechanism of TCM (Guo et al., 2023; Li et al., 2023). The rat adrenal medulla pheochromocytoma cell line PC12, exhibiting typical neuronal characteristics and possessing a high level of GCs receptors, serves as a primary cell model for *in vitro* neurobiological and neurochemical research (Mao et al., 2011). It has been extensively utilized as a neuroprotective *in vitro* research model to simulate hippocampal neuron damage induced by high concentrations of Corticosterone (Cort) stimulation (Zhou et al., 2017).

In this study, we aimed to screen for potential neuroprotective active components, target molecules, and crucial signaling pathways in HLTP by network pharmacology. Molecular docking is utilized to analyze the binding abilities of predicted and already proven neuroprotective-related targets. By establishing a Cort-induced PC12 cell injury model, the study investigates whether HLTP possess neuroprotective effects on nerve cells. The study validates the predicted pathways through Western blot experiments. Ultimately, this demonstrates the neuroprotective effect of HLTP, aiming to provide a research foundation and basis for the preventive treatment of depression with *H. citrina* leaves.

## 2 Materials and methods

### 2.1 Reagents and materials

*Hemerocallis citrina* leaves were collected in Qingyang City, Gansu Province, and identified as fresh leaves of the lily family plant *H. citrina* Baroni by Chief Pharmacist Yang Xicang at the Affiliated Hospital of Gansu University of Chinese Medicine. The differentiated PC12 cells were generously provided by Professor Zhi Dejuan from Lanzhou University.

RPMI 1640 medium (SH30809.01) and PBS solution (SH30256.01) were purchased from Cytiva. Efficient RIPA lysis buffer (R0010), Penicillin-streptomycin dual antibodies (P1400), fetal bovine serum (S9020), horse serum (S9050), trypsin (T1300), mitochondrial membrane potential detection kit (M8650), SDS-PAGE gel preparation kit (P1200), and ECL chemiluminescence reagent (PE0010) were obtained from Solarbio. MTT (M405849) and Corticosterone (C104537) were purchased from Aladdin. LDH (A020-2-2), MDA (A003-1-2), SOD (A001-3-2), CAT (A007-1-1), Annexin V-FITC (G003-1-3), and Hoechst33342/PI (G023-1-1) detection kits were purchased from Nanjing Jiancheng Bioengineering Institute. ROS (S0033S) and Ca^2+^ (S1061S) detection kits were obtained from Beyotime. Antibodies Bax (YT0455), Bcl-2 (YT0470), Caspase-3 (YC0026), Cyt-C (YM3402), TNF-α (YT4689), IL-1β (YM4682), IL-10 (YT5138), AKT (YP0006), β-actin (YM3028), HRP*Goat Anti Rabbit IgG (H + L) (RS0002) and HRP*Goat Anti Mouse IgG (H + L) (RS0001) were purchased from immunoway. Antibodies PI3K (4292) was purchased from Cell Signaling Technology. Standard substances for Quercetin (purity ≥ 98%, 200449-3), Rutin (purity ≥ 99%, 103082), Apigenin (purity ≥ 98%, 102546), Isoquercitrin (purity ≥ 98%, 200037-1), 4-O-Caffeoylquinic acid (purity ≥ 98%, 103729), 4-p-Coumaroylquinic acid (purity ≥ 97%, 104083-221102) and 5-O-Feruloylquinic acid (purity ≥ 99%, 103588) were procured from Jiangsu Yongjian Pharmaceutical. Standard substances for 5-O-Caffeoylshikimic acid (purity ≥ 98%, DSTDK008901) was purchased from Chengdu DeSiTe Biological. Organic solvents such as methanol, acetonitrile, and formic acid are chromatographically and mass spectrometry pure (Beijing Bailingwei Technology Co., Ltd.), and water is Watsons purified water.

### 2.2 Preparation of HLTP

The 5.0 g *H. citrina* leaves powder was accurately weighed, and 75 mL of 80% ethanol was added to heat reflux extraction for 1 h, and the residue was added to 50 mL of 80% ethanol for reflux extraction for 1 h. The filtrate was combined twice and concentrated to no alcohol flavor under reduced pressure. The concentrate was placed in an evaporation dish and dried to obtain the extract. The extract was weighed and distilled water was added to prepare a 3 mg/mL test solution. 35 mL of the test solution was taken and subjected to DM-21 macroporous adsorption resin column chromatography. Elution was performed with 135 mL of 60% ethanol at a flow rate of 2 mL/min, and the eluent was collected and concentrated to an appropriate volume under reduced pressure. Freeze-dried into freeze-dried powder for use and dissolved in an appropriate amount of distilled water to obtain a certain concentration of HLTP. 5 mL of HLTP was extracted twice with 5 mL of n-butanol. The extract was combined twice and concentrated to dry under reduced pressure, diluted to 5 mL with methanol solution, filtered through a 0.22 μm organic filter membrane to obtain the sample solution for UPLC-Q-TOF-MS analysis.

### 2.3 Identification of chemical constituents of HLTP by UPLC-Q-TOF-MS

#### 2.3.1 Chromatographic condition

The study was performed on a Waters H-Class UPLC system. Chromatographic separation was achieved on a Waters ACQUITY UPLC®HSS T3 (2.1 mm × 100 mm, 1.8 μm), protected by VanGuandTM BEHC C18 at 40. The mobile phase was composed of ultra-pure water with 0.1% formic acid (A) and acetonitrile (B) at a flow rate of 0.4 mL/min. The gradient elution profile was as follows: 0–4 min, 5% B; 4∼9 min, 5%–10% B; 9∼24 min, 10%–20% B; 24∼26 min, 20%–25% B; 26∼28 min, 25%–40% B; 28∼34 min, 40%–90% B; 34∼37 min, 90% B; 37∼39 min, 90%–5% B. The injection volume was 2 μL. The temperature of the autosampler was kept at 4°C.

#### 2.3.2 Mass spectrometer conditions

The UPLC-MS/MS analysis on HLTP extract was performed on Waters H-Class UPLC system coupled with an Agilent 6,560 quadrupole time-of-flight tandem mass spectrometer. MS analysis was performed at electrospray ionization in negative and positive ion mode and the acquisition parameters were as follows: drying gas, N_2_; drying gas flow rate, 10 L/min; drying gas temperature, 225°C; nebulizer pressure, 25 psi; Electrospray ion source with capillary voltages of 3,500 V and 4,000 V in positive and negative ion modes, respectively; scan range m/z 20∼1,700; fragmentor voltage, 80 V; collision energy, 10, 20, 40 V; Data acquisition was performed using a Masshunter workstation.

### 2.4 Network pharmacology analysis

#### 2.4.1 Prediction of potential components and targets for the neuroprotective effects of *Hemerocallis citrina* leaves

This study first queried the SMILES numbers of the 32 identified phenolic components in *H. citrina* leaves through the DrugBank database (http://www.drugbank.ca), as shown in Table 1. Compounds not found in the database were drawn using ChemDraw and converted to SMILES format using the OpenBabel molecular format conversion tool. The SMILES numbers were uploaded to the Swiss Target Prediction database (http://www.swisstargetprediction.ch), with the attribute set to “Homosapiens,” to obtain target information. Using “Neuroprotection” as a keyword, we retrieved databases such as GenCards (https://www.genecards.org/) and OMIM database (https://omim.org/) to search target information. Removing the same targets and using Venny 2.1 (https://bioinfogp.cnb.csic.es/tools/venny/index.html) to obtain common targets. The obtained common targets were uploaded to the STRING database (https://www.string-db.org/) to retrieve the PPI network. Using Cytoscape 3.9.1 software, we drew the network diagram of “*H. citrina* leaves Phenolic Components - Common Targets - Neuroprotection” and selected core chemical components based on the Degree value, Betweenness Centrality, and Closeness Centrality of active ingredients.

**TABLE 1:** Chemical Information of Flavonoid Components in *Hemerocallis citrina* Baroni leaves.

Number	Molecular formula	Chemical name	Structure
M1	C_16_H_18_O_9_	Chlorogenic acid	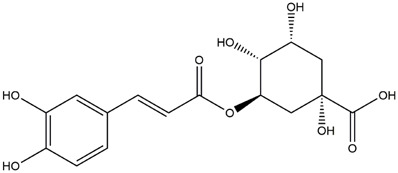
M2	C_16_H_18_O_9_	Neochlorogenic acid	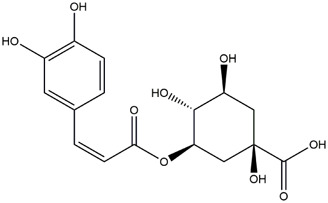
M3	C_16_H_18_O_8_	3-p-Coumaroylquinic acid	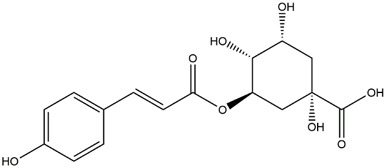
M4	C_16_H_18_O_9_	1-O-Caffeoylquinic acid	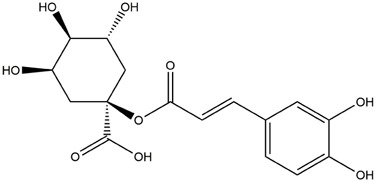
M5	C_17_H_20_O_9_	3-O-Feruloylquinic acid	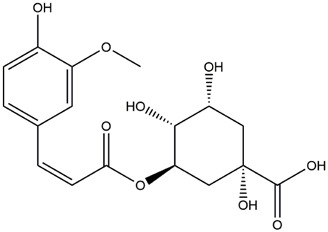
M6	C_16_H_18_O_9_	4-O-Caffeoylquinic acid	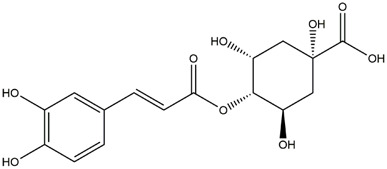
M7	C_16_H_18_O_8_	5-p-Coumaroylquinic acid	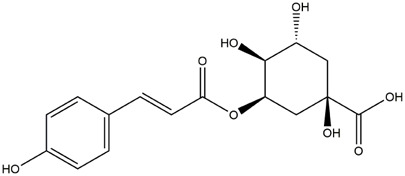
M8	C_16_H_18_O_8_	4-p-Coumaroylquinic acid	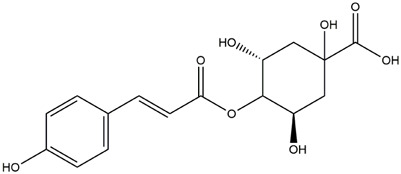
M9	C_16_H_16_O_8_	5-O-Caffeoylshikimic acid	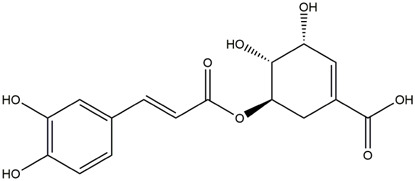
M10	C_17_H_20_O_9_	5-O-Feruloylquinic acid	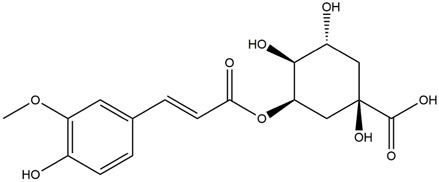
M11	C_27_H_30_O_15_	Vicenin-2	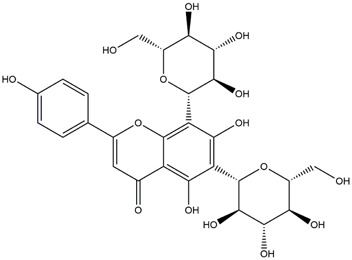
M12	C_16_H_16_O_8_	3-O-Caffeoylshikimic acid	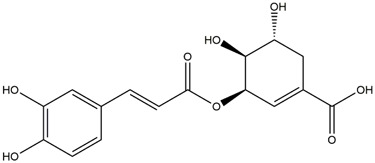
M13	C_16_H_16_O_8_	4-O-Caffeoylshikimic acid	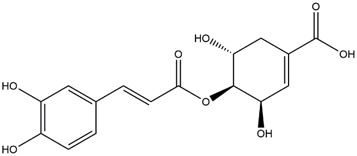
M14	C_33_H_40_O_20_	Quercetin-3-O-α-L-rhamnosyl-(1→6)-[α-L-rhamnosyl-(1→2)]-β-D-glucoside	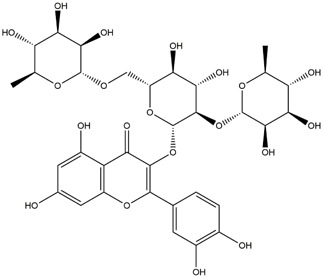
M15	C_33_H_40_O_20_	Quercetin-3-O-α-L-rhamnosyl-(1→6)-β-D-D-glucosyl-(1→2)-α-L-rhamnoside	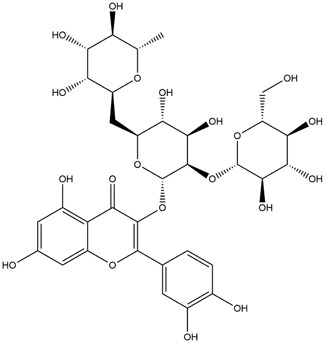
M16	C_27_H_30_O_16_	Quercetin-3-O-α-L-rhamnosyl-(1→6)-β-D-galactoside	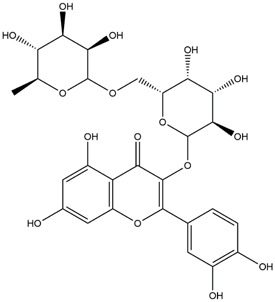
M17	C_27_H_30_O_16_	Rutin	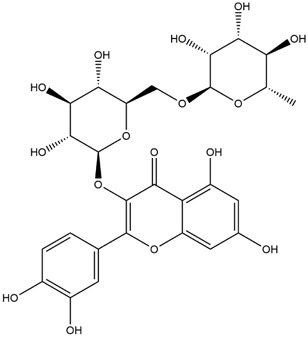
M18	C_33_H_40_O_19_	Kaempferol-3-O-rhamnoglucosyl-7-O-rhamnoside	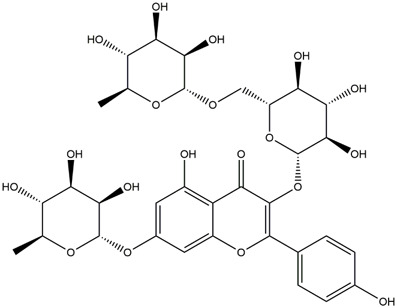
M19	C_34_H_42_O_20_	Isorhamnetin-3-O-rutinoside-7-O-rhamnoside	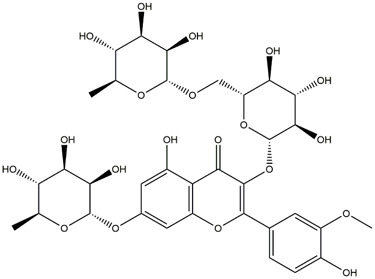
M20	C_21_H_20_O_12_	Hyperoside	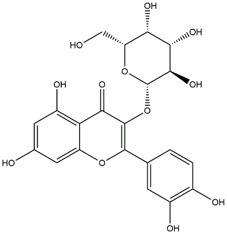
M21	C_21_H_20_O_12_	Isoquercitrin	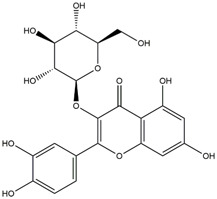
M22	C_21_H_22_O_11_	Dihydrokaempferol-7-O-β-D-glucoside	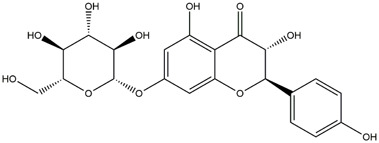
M23	C_28_H_32_O_16_	Isorhamnetin-3-O-β-D-rutinoside	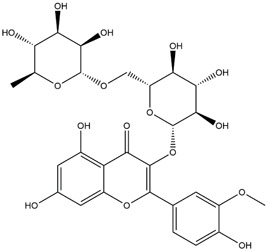
M24	C_26_H_28_O_15_	Quercetin-3-O-α-L-rhamnose-(1→2)-α-L-arabinopyranoside	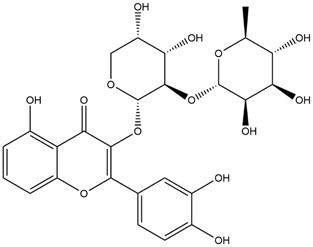
M25	C_20_H_18_O_11_	Quercetin-3-O-α-L-arabinopyranoside	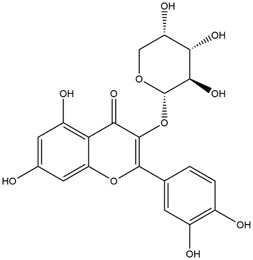
M26	C_21_H_20_O_11_	Kaempferol-3-O-α-D-glucoside	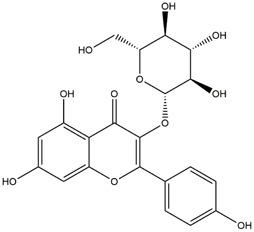
M27	C_21_H_20_O_11_	Quercetin 3-O-α-L-rhamnoside	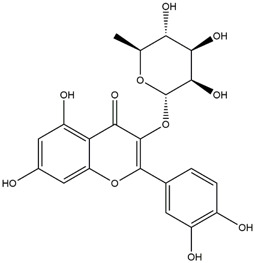
M28	C_27_H_30_O_15_	Quercetin-3,7-O-L-Dirhamnoside	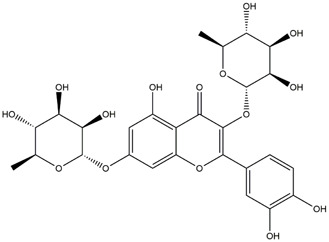
M29	C_27_H_30_O_15_	Isorhamnetin-3-rhamnose-(1→2)-α-L-arabinopyranoside	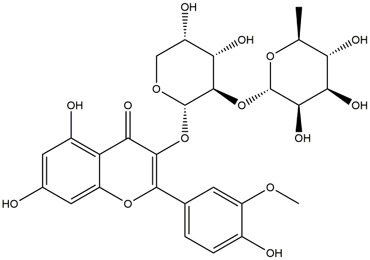
M30	C_28_H_32_O_15_	diosmetin-7-O-rutinoside	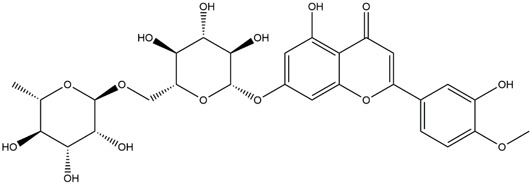
M31	C_15_H_10_O_7_	Quercetin	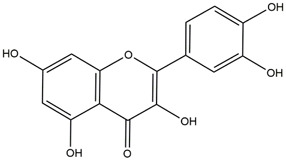
M32	C_15_H_10_O_5_	Apigenin	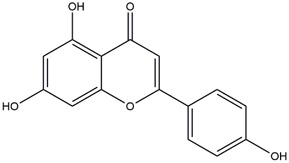

#### 2.4.2 GO function enrichment analysis and KEGG pathway enrichment analysis

The common targets were imported into the Metascape database (https://metascape.org/) for GO function and KEGG pathway enrichment analysis. GO analysis yielded three parts: Biological Process (BP), Cellular Component (CC), and Molecular Function (MF), and generated bar charts. The top 20 signal pathways related to neuroprotection were visualized in a functional enrichment bubble chart based on the enriched KEGG pathways. Using the selected common targets and enriched KEGG pathways, an analytical network of “*H. citrina* leaves Phenolic Components - Common Targets - Pathways” was constructed using Cytoscape 3.9.1 software.

#### 2.4.3 Molecular docking analysis

Autodock software was used to perform molecular docking between the targets associated with neuroprotection (including screened targets and the targets reported in the literature related to neuroprotection) and *H. citrina* leaves phenolic components. The binding activities between active components of *H. citrina* leaves and common targets were compared. The results with optimal conformations and energy were selected for further analysis, and the PyMOL software was used to interpret and visualize the macromolecule-ligand complexes.

#### 2.4.4 *In vitro* neuroprotective activity screening of major phenolic components in *Hemerocallis citrina* leaves

Cort-induced PC12 cell injury model was used for *in vitro* screening of neuroprotective activity of the core components selected through network pharmacology and molecular docking (specific PC12 cell culture conditions and dosing methods are detailed in Section 2.5). For the screening of the core phenolic components of *H. citrina* leaves, the concentrations were set at 1, 10, 20, and 50 μmol/L based on literature (Zhou et al., 2017). According to the results, concentrations that had no impact on cell viability were selected for the activity screening experiment of phenolic components against Cort-induced PC12 cell damage.

### 2.5 Neuroprotective activity evaluation

#### 2.5.1 Construction of cell injury model

PC12 cells were cultured with RPMI 1640 complete medium (containing 5% fetal bovine serum, 5% horse serum, and 1% penicillin-streptomycin) in a 37°C, 5% CO_2_ cell culture incubator. When the cells density reached 80%∼90%, they were passaged with 0.25% trypsin, and logarithmic phase cells were selected for the experiment. PC12 cells were seeded in a 96-well plate at a density of 5×10^4^/mL, with 0.2 mL per well, and cultured for 24 h. The control group cells were cultured with complete medium. The experimental group cells were treated with Cort at final concentrations of 50, 100, 200, 400, and 800 μmol/L for 24 h. After removing the culture medium, 200 μL of MTT (0.5 mg/mL) were added to each well, and after 4 h of incubation, the supernatant was removed, and 150 μL of DMSO was added to each well. Shake in the dark for 15 min on a microplate shaker, and the absorbance A value of each well was measured at 470 nm by a microplate reader. The cell viability was calculated as follows: Cell viability = (A model/A control) × 100%.

#### 2.5.2 Determination of cell viability assay and LDH release

PC12 cells were seeded in a 96-well plate at a density of 5×10^4^/mL, with 0.2 mL per well, and cultured for 24 h. Control and experimental groups were established. The control group cells were cultured with complete medium. The experimental groups cells were treated with HLTP of 50, 100, 200, 500, and 1,000 μg/mL, referring to the research of Tian et al. (2017), and the cell viability was detected by the above MTT method to screen HLTP concentration. PC12 cells were seeded at a density of 5 × 10^4^/mL in a 96-well plate with 0.2 mL per well and cultured for 24 h. Cells were divided into control group, model group, low-dose group, medium-dose group, and high-dose group of HLTP. The control group added 200 μL of complete medium, the model group and HLTP groups added 200 μL of complete medium with the optimal concentration of Cort. After 24 h, the entire culture medium was aspirated, and the control and model groups added 200 μL of fresh medium. The HLTP groups added 200 μL of medium containing low, medium, and high concentrations of HLTP. After 24 h of incubation, cell viability was determined using the MTT assay mentioned above. The LDH content was determined following the instructions of the LDH assay kit. The LDH level in the extracellular fluid reflects the degree of cell damage.

#### 2.5.3 Determination of SOD, MDA, ROS, and CAT levels

PC12 cells were seeded in a 6-well plate at a density of 4 × 10^5^/mL, with 2 mL per well, and cultured for 24 h. Cells were treated with the experimental grouping and cell treatment methods described in Section 2.5.2. The cell supernatant was discarded, trypsinized and resuspended with PBS. Then, cells were treated with cell disruptor, and the supernatant was taken after centrifugation. Commercial assay kits were used to determine cellular MDA content, total SOD levels, CAT activity, and ROS levels. ROS changes were also measured using a fluorescence spectrophotometer, and the enzyme levels in cells were expressed as a percentage of the control group.

#### 2.5.4 Determination of cell apoptosis rate, Ca^2+^, and MMP by fluorescence microscopy and flow cytometry

PC12 cells were seeded at a density of 4 × 10^5^/mL cells/mL in a 6-well plate with 2 mL per well and cultured for 24 h. Cells were treated according to the experimental grouping and cell processing methods mentioned Section 2.5.2. The treated cells were aspirated of the culture medium, washed twice with PBS. Then, 0.5 mL of Hoechst 33258 staining solution was added, stained at 37°C in the dark for 20 min, washed twice with PBS. 0.5 mL of PI staining solution was added, stained at 37°C in the dark for 20 min, washed twice with PBS. The morphology of cells was observed under a fluorescence microscope. The treated cells were aspirated of the culture medium, washed twice with PBS, resuspended in 200 μL Binding Buffer, and then transferred 200 μL of cell suspension from each group to a flow tube. Subsequently, 5 μL of Annexin V-FITC solution and 5 μL of PI staining solution were added, mixed well, and kept in the dark at room temperature for 10 min before detection and analysis using a flow cytometer. The treated cells were aspirated of the culture medium, and each group was made into 0.5 mL of cell suspension, added 10 μmol of Fura-3/AM (5 μmol/L), and incubated at 37°C for 30 min. After washing twice with D-hanks buffer, the cells were further incubated for 30 min. A 1 mL cell suspension was prepared using D-hanks buffer, and the fluorescence intensity was measured by flow cytometry. Simultaneously, the cells were placed on the slide and observed under a fluorescence microscope. The treated cells were aspirated of the culture medium, washed twice with PBS, and the JC-1 staining working solution was diluted with culture medium at a ratio of 1:1. Then, 2 mL of staining solution was added to each well, incubated at 37°C for 20 min in the cell culture incubator. After aspirating the supernatant, cells were washed twice with JC-1 staining buffer, and the mitochondrial membrane potential (MMP) was measured using a flow cytometer.

#### 2.5.5 Determination of the protein levels of Bax, Caspase-3, Bcl-2, Cyt-C, β-actin, IL-1β, TNF-α, IL-10, PI3K and AKT by Western blotting

PC12 cells were seeded at a density of 4 × 10^5^ cells/mL in a 6-well plate with 2 mL per well and cultured for 24 h. Cells were treated according to the experimental grouping and cell processing methods mentioned Section 2.5.2. The culture medium was aspirated, washed twice with PBS. Cells were then lysed on ice with RIPA lysis buffer containing a protease inhibitor. After protein extraction, the protein samples were denatured by heating at 99°C for 10 min. The protein samples were subjected to 10% SDS-PAGE, transferred to a PVDF membrane, and blocked in 5% skimmed milk for 2 h. The membrane was then incubated with specific antibodies (Bax, Caspase-3, Bcl-2, Cyt-C, β-actin, IL-1β, TNF-α, IL-10, PI3K, AKT) overnight at 4°C, washed with PBS three times, each 10 min, incubated with HRP-conjugated goat anti-rabbit secondary antibody at room temperature for 2 h, washed with PBS 3 times, visualized by ImageJ software with grayscale analysis.

### 2.6 Statistical analysis

Statistical analysis was performed using SPSS 19.0 software, and data are presented as means ± SD. Between-group comparisons were conducted using one-way ankalysis of variance (ANOVA), and multiple tests were conducted using Tukey’s multiple comparisons test. The analysis of ANOVA results was presented as F (numerator degrees of freedom (DFn), denominator degrees of freedom (DFd)), with *P* < 0.05 was indicated significant differences.

## 3 Results

### 3.1 Identification of chemical constituents of HLTP

The total ion chromatogram of HLTP under UPLC-Q-TOF-MS negative ion mode is shown in Supplementary Figure S1. Based on the fragmentation pattern of mass spectrometry and relevant literature reports, a total of 32 components were identified, including 12 phenolic acids and 20 flavonoids. The retention time, mass charge ratio, and fragmentation information of each chemical component are shown in Supplementary Table S1.

### 3.2 Network pharmacology analysis results

#### 3.2.1 Potential components and targets for the neuroprotective effects of *Hemerocallis citrina* leaves

**FIGURE 1:**
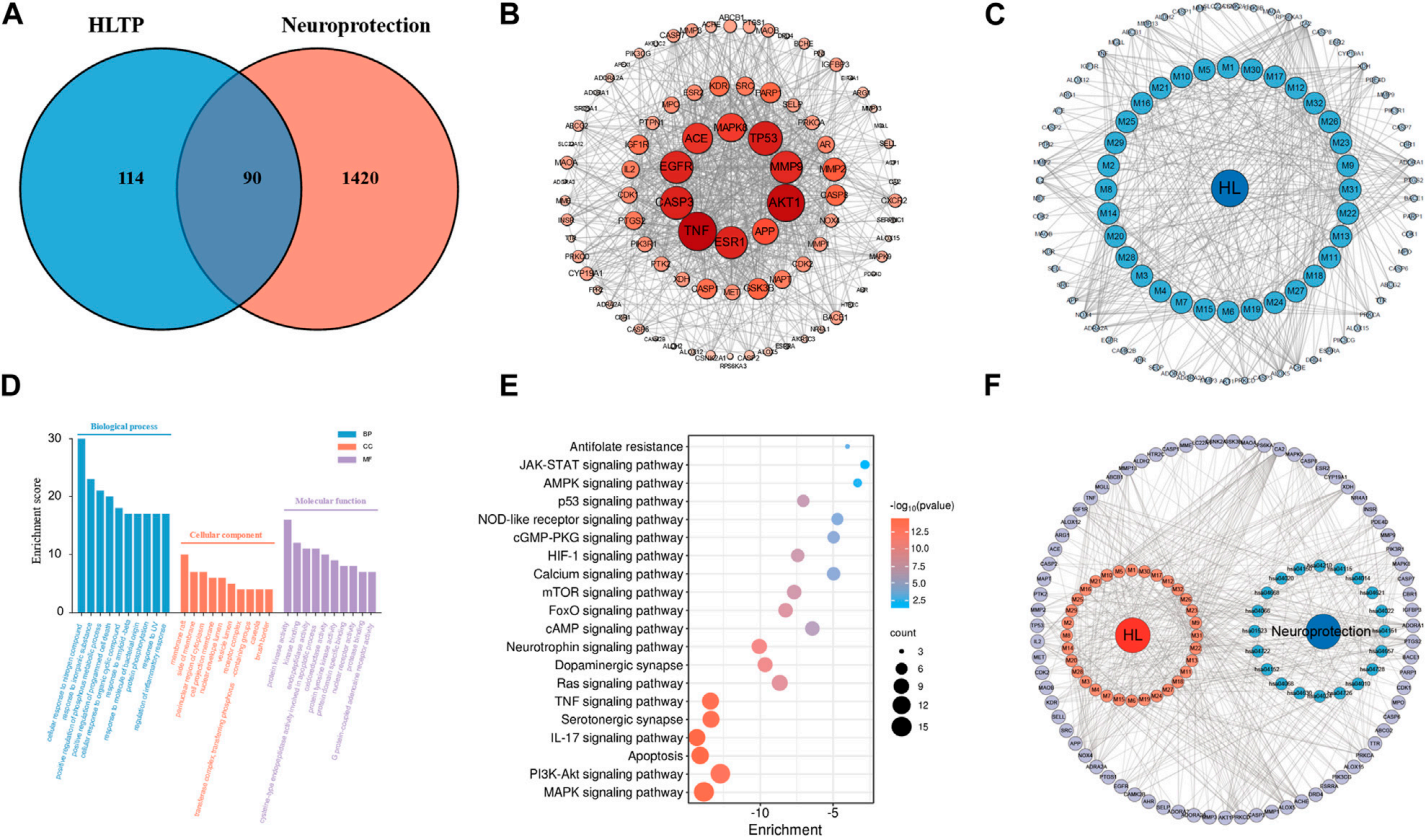
Network pharmacology analysis of the neuroprotective effects of phenolic components in *H. citrina* leaves (**(A)** Venn diagram of phenolic components in *H. citrina* leaves and neuroprotective targets; **(B)** PPI network; **(C)** Network of “*H. citrina* leaves Phenolic Components - Common Targets – Neuroprotection”; **(D)** GO biological function enrichment; **(E)** KEGG pathway enrichment; **(F)** Network of “*H. citrina* leaves Phenolic Components - Common Targets - Pathways.” The component targets and disease targets information were searched through databases, and result graph were drew by Cytoscape software and Microbioinformatics Platform (https://www.bioinformatics.com.cn/)).

Based on 32 components of *H. citrina* leaves, 204 unique component targets were obtained from the Swiss Target Prediction database. Using databases such as GenCards and OMIM, 1,510 neuroprotective-related targets were excavated. After Venn analysis, 90 common targets were identified, representing potential targets for the neuroprotective effects of *H. citrina* leave phenolic compounds, as shown in Figure 1A. The PPI network relationship diagram was constructed, as shown in Figure 1B. The network diagram consists of 90 nodes and 836 edges, with an average Degree of 18.6. Node size and color depth are positively correlated with the Degree values of the selected neuroprotective target proteins. Larger and darker nodes such as TNF, AKT1, TP53, CASP3, EGFR, ESR1, MMP9, ACE, MAPK8, and APP may play a crucial role in neuroprotection. The “*H. citrina* leaves Phenolic Components-Common Targets-Neuroprotection” network diagram was constructed, as shown in Figure 1C. This network diagram comprises 122 nodes, where light blue nodes represent the 32 components, and white nodes represent the 90 neuroprotective-related targets. Based on the Degree, Betweenness Centrality, and Closeness Centrality network topology analysis of active components (Supplementary Table S2), four core chemical components were selected: M32 (Apigenin), M31 (Quercetin), M17 (Rutin), and M21 (Isoquercitrin), which may be the main active components responsible for the neuroprotective effects of *H. citrina* leaves phenolic components.

#### 3.2.2 Potential signaling pathways for the neuroprotective effects of *Hemerocallis citrina* leaves

The GO enrichment analysis resulting in 288 entries related to BP, 75 to CC, and 115 to MF. The top ten enriched entries were visualized, as shown in Figure 1D. In BP analysis, it mainly involves cellular response to organonitrogen compound, positive regulation of phosphorylation, and regulation of inflammatory response. In CC analysis, it mainly involves membrane raft, perinuclear region of cytoplasm, vesicle lumen, etc. In MF analysis, it mainly involves nuclear receptor activity, oxidoreductase activity, kinase binding, etc. These may be related to the complex pathogenesis of neural damage, suggesting that *H. citrina* leaves phenolic compounds can exert neuroprotective effects through different levels and multiple pathways. The KEGG pathway enrichment resulting in 179 pathway entries. These pathways involve various diseases such as cancer, lipid metabolism, atherosclerosis, and diabetic cardiomyopathy. The top 20 pathways related to neuroprotection were selected, as shown in Figure 1E. The main pathways involve the MAPK pathway, PI3K/AKT pathway, apoptosis pathway, and IL-17 pathway. The “*H. citrina* leaves Phenolic Components-Common Targets-Pathways” was constructed, as shown in Figure 1F. Based on comprehensive PPI analysis and KEGG enrichment analysis, it is speculated that *H. citrina* leaves phenolic components may exert neuroprotective effects by regulating the PI3K/AKT pathway and its downstream pathways.

#### 3.2.3 Molecular docking results

**FIGURE 2:**
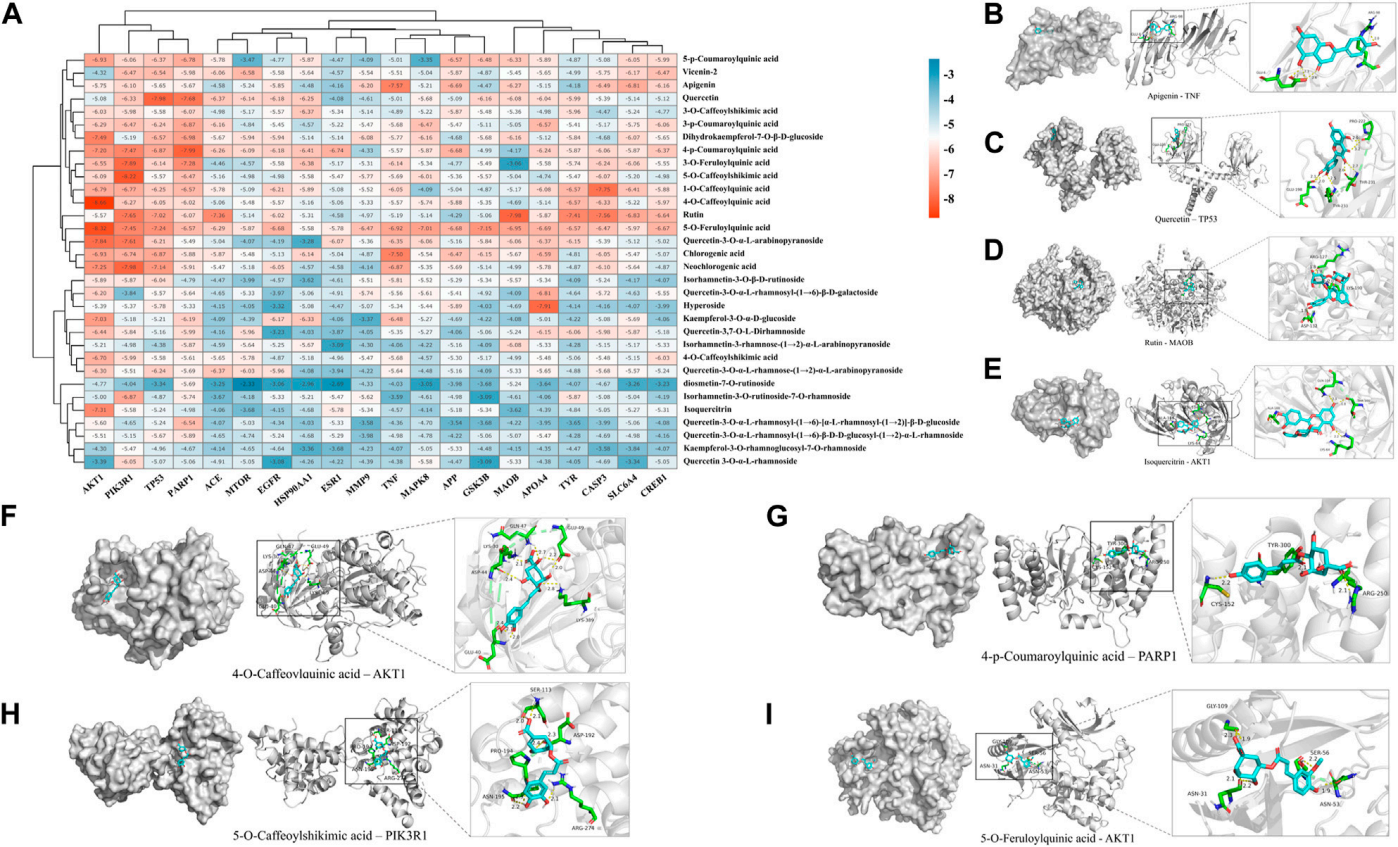
Molecular docking of the neuroprotective effects of phenolic components in *H. citrina* leaves with core target proteins and core components **(A)** Docking result heatmap; **(B)** Docking result between Apigenin and TNF; **(C)** Docking result between Quercetin and TP53; **(D)** Docking result between Rutin and MAOB; **(E)** Docking result between Isoquercitrin and AKT1; **(F)** Docking result between 4-O-Caffeoylquinic acid and AKT1; **(G)** Docking result between 4-p-Coumaroylquinic acid and PARP1; **(H)** Docking result between 5-O-Caffeoylshikimic acid and PIK3R1; **(I)** Docking result between 5-O-Feruloylquinic acid and AKT1. Autodock software was used to perform molecular docking analysis on the screened targets and 32 components, and PyMOL software was used to visualize the protein with the strongest binding to the screened components.

To validate the results of network pharmacology, we conducted molecular docking verification between the 32 phenolic components in *H. citrina* leaves with the proteins. These proteins including top ten target proteins (TNF, AKT1, TP53, CASP3, EGFR, ESR1, MMP9, ACE, MAPK8, and APP) and proteins that ranked lower or did not appear in the screening results but have been extensively studied and proven to be related to neuroprotection or serve as targets for some neuroprotective drugs (GSK3B, PIK3R1, MTOR, MAOB, TYR, APOA4, SLC6A4, PARP1, HSP90AA1, CREB1). The results are shown in Figure 2A. Generally, when the binding energy is <−5.0 kcal/mol, it indicates a strong binding activity between the compound and the target. Overall, most components exhibit good binding affinity with proteins. The phenolic acid components in *H. citrina* leaves generally have lower binding energies compared to flavonoid components, indicating better binding. Among all neuroprotective-related target proteins, the phenolic components that bind well to AKT1, PIK3R1, TP53, and PARP1 are the most abundant. Further visualization analysis was performed on the four components with the strongest binding to AKT1, PIK3R1, TP53, and PARP1, as well as the four core chemical components selected through comprehensive screening of network topology analysis. The results are shown in Figures 2B–I. Apigenin has good binding sites with TNF, Quercetin with TP53, Rutin with MAOB, Isoquercitrin with AKT1, 4-O-Caffeoylquinic acid with AKT1, 4-p-Coumaroylquinic acid with PARP1, 5-O-Caffeoylshikimic acid with PIK3R1, and 5-O-Feruloylquinic acid with AKT1. For instance, 4-O-Caffeoylquinic acid binds to a deep groove on the surface of the receptor protein AKT1, and they have good shape complementarity. The binding is mainly stabilized through hydrogen bonding, and the residues involved in hydrogen bonding with 4-O-Caffeoylquinic acid include LYS389, GLU49, GLN47, LYS30, ASP44, and GLU40.

#### 3.2.4 *In vitro* neuroprotective activity of main phenolic components in *Hemerocallis citrina* leaves

**FIGURE 3:**
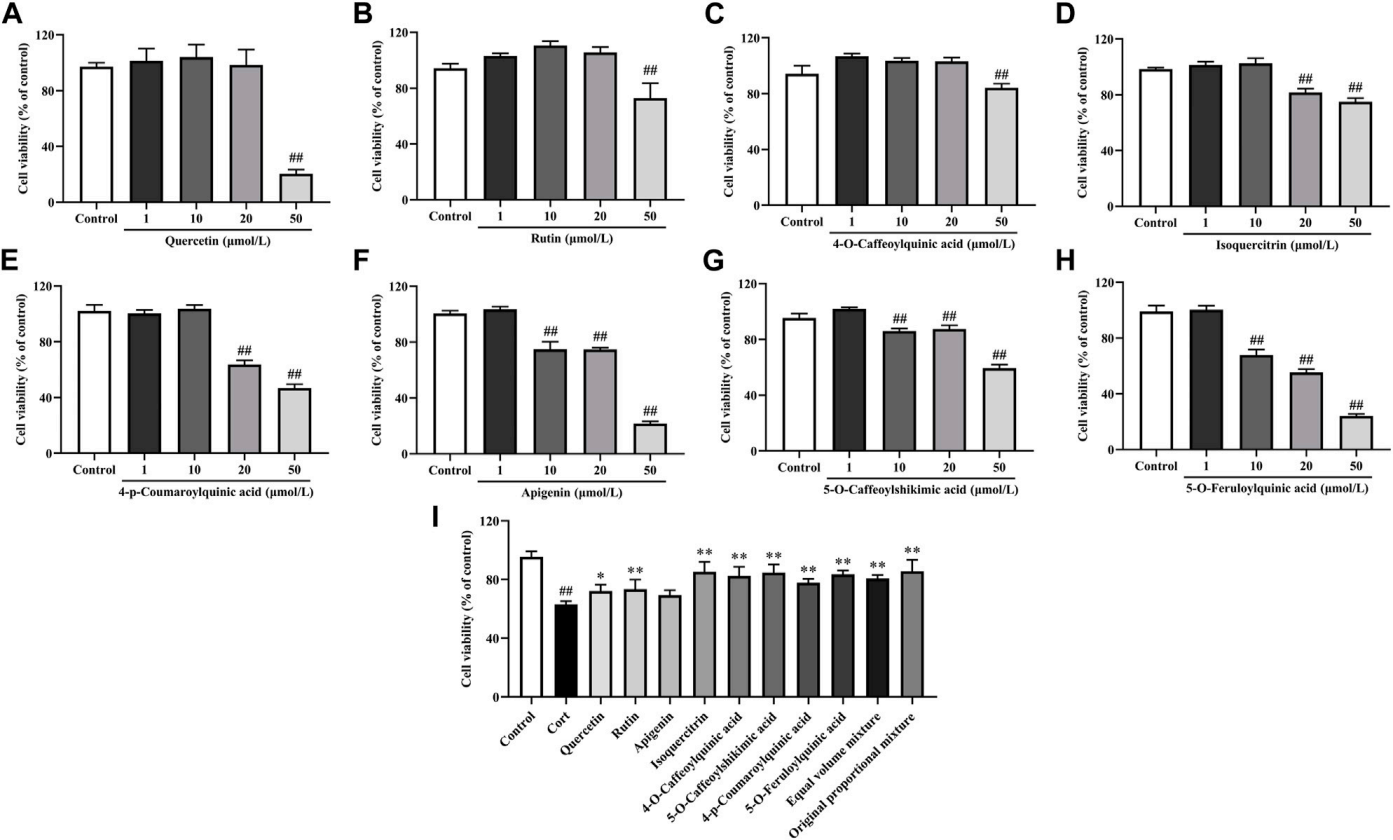
*In vitro* neuroprotective activity of main phenolic components in *H. citrina* leaves **(A)** Quercetin, *F* = 112.4, *P* < 0.0001; **(B)** Rutin, *F* = 39.0, *P* < 0.0001; **(C)** 4-O-Caffeoylquinic acid, *F* = 84.9, *P* < 0.0001; **(D)** Isoquercitrin, *F* = 120.3, *P* < 0.0001; **(E)** 4-p-Coumaroylquinic acid, *F* = 566.0, *P* < 0.0001; **(F)** Apigenin, *F* = 744.9, *P* < 0.0001; **(G)** 5-O-Caffeoylshikimic acid, *F* = 383.7, *P* < 0.0001; **(H)** 5-O-Feruloylquinic acid, *F* = 815.2, *P* < 0.0001; **(I)** Influence of the main phenolic components in *H. citrina* leaves and their equimolar mixture on the viability of PC12 cells induced by Cort, *F* = 20.2, *P* < 0.0001) (Note: Compared with the Control group: ^#^ *P* < 0.05 and ^##^ *P* < 0.01; Compared with the Model group: *P< 0.05 and **P< 0.01; mean ± SD, *n* = 5; **(A–H)** DFn = 4, DFd = 20; **(I)** DFn = 11, DFd = 48. Screening the optimal drug concentrations of eight components using MTT method and their neuroprotective effects on Cort induced PC12 cells).

The research results of the main phenolic components in *H. citrina* leaves on PC12 cells are shown in Figure 3. After the treatment of PC12 cells with 50 μmol/L Quercetin, Rutin, and 4-O-Caffeoylquinic acid, 20∼50 μmol/L Isoquercitrin and 4-p-Coumaroylquinic acid, and 10∼50 μmol/L Apigenin, 5-O-Caffeoylshikimic acid, and 5-O-Feruloylquinic acid for 24 h, the cell viability significantly decreased, all below 90%. However, after treating cells with remaining concentrations of individual phenolic components for 24 h, the cell viability was all above 95%, indicating no effect on the cells. Therefore, in the active screening experiment, the concentrations selected for Quercetin, Rutin, Isoquercitrin, 4-O-Caffeoylquinic acid, and 4-p-Coumaroylquinic acid were 10 μmol/L, while those for Apigenin, 5-O-Caffeoylshikimic acid, and 5-O-Feruloylquinic acid were 1 μmol/L. The research results on the protective effect of the main phenolic components in *H. citrina* leaves against Cort-induced damage to PC12 cells are shown in Figure 3I. Compared to the Control group, the cell viability significantly decreased after Cort (200 μmol/L, the concentration screening process is shown in Section 3.3) treatment of PC12 cells (*P* < 0.01). Subsequently, treating cells with the eight main individual components in *H. citrina* leave, as well as their equimolar mixture and the original proportion mixture, for 24 h resulted in a significant increase in cell viability for all components except Apigenin (*P* < 0.05 or *P* < 0.01), demonstrating a pronounced protective effect. Additionally, the study found that the original proportion mixture of the eight main individual components in *H. citrina* leave has a stronger improvement effect on the cell viability of PC12 cells. Therefore, subsequent *in vitro* activity evaluations chose HLTP as a therapeutic drug.

### 3.3 *In vitro* activity evaluation results

#### 3.3.1 Impact of HLTP on cell damage and the levels of MDA, SOD, CAT, and ROS

**FIGURE 4:**
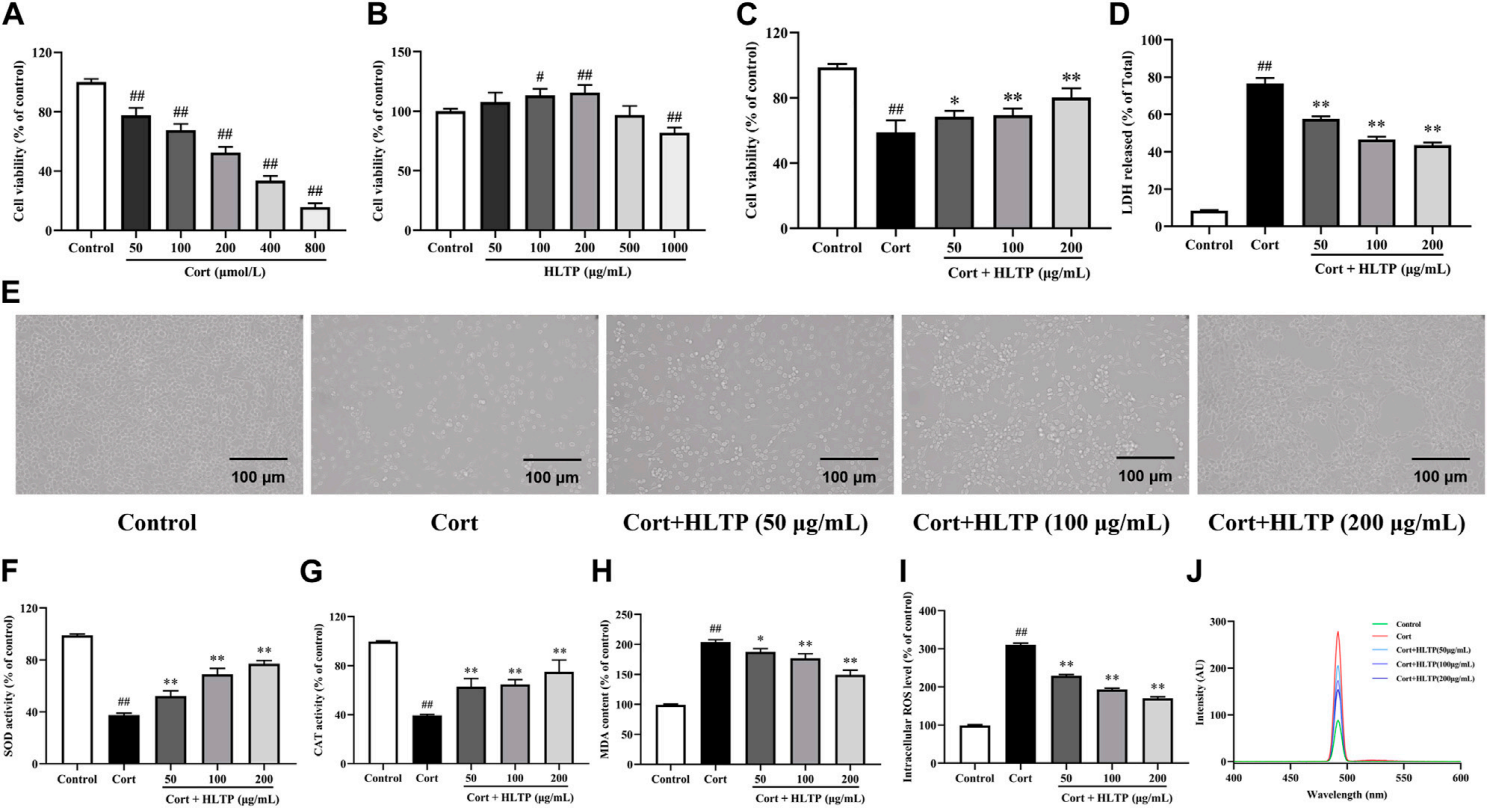
Effects of HLTP on the degree of cell damage and the levels of intracellular oxidases and antioxidants (**(A)** Effect of Cort on cell viability, *F* = 354.0, *P* < 0.0001; **(B)** Effect of HLTP on cell viability, *F* = 21.4, *P* < 0.0001; **(C)** Effect of HLTP on viability of damaged cells, *F* = 56.8, *P* < 0.0001; **(D)** Effect of HLTP on cell LDH, *F* = 635.2, *P* < 0.0001; **(E)** Effect of HLTP on the morphology of Cort-damaged PC12 cells; **(F)** SOD level, *F* = 178.5, *P* < 0.0001; **(G)** CAT level, *F* = 47.1, *P* < 0.0001; **(H)** MDA level, *F* = 148.9, *P* < 0.0001; **(I, J)** ROS levels, *F* = 1,496.0, *P* < 0.0001) (Note: Compared with the Control group: ^#^ *P* < 0.05 and ^##^ *P* < 0.01; Compared with the Model group: *P < 0.05 and **P < 0.01; mean ± SD; **(A, B)**: *n* = 5, DFn = 5, DFd = 24; **(C, D)** and **(F–I)** *n* = 3, DFn = 4, DFd = 10. MTT method was used to screen the concentrations of HLTP and Cort, while detecting the protective effect of HLTP on damaged cells and their effects on oxidase and antioxidant enzymes).

Firstly, the impact of Cort and HLTP at different concentrations on cell viability was analyzed, as shown in Figures 4A, B. After stimulating PC12 cells with 200 μmol/L Cort for 24 h, the cell viability was close to 50% (*P* < 0.01). Therefore, 200 μmol/L Cort stimulation for 24 h was chosen as the experimental condition for the neuronal cell damage model. HLTP (50, 100, and 200 μg/mL) had no significant effect on PC12 cell viability. When the HLTP concentration reached 1,000 μg/mL, the cell viability significantly decreased (*P* < 0.01). Therefore, the subsequent concentration of HLTP was controlled within 200 μg/mL. The impact of HLTP on the cell damage model was analyzed, as shown in Figure 4C. Compared to the Control group, the cell viability of the Model group significantly decreased (*P* < 0.01), with a reduction of 39.77%. After administering HLTP (50, 100, and 200 μg/mL), the cell viability significantly increased (*P* < 0.05 or 0.01) 9.50%, 10.42%, and 21.25%, respectively. It is suggested that HLTP have a good protective effect against Cort-induced neuronal damage. The LDH detection results are shown in Figure 4D. The addition of HLTP (50, 100, and 200 μg/mL) significantly reduced cell LDH release rates (*P* < 0.01). Compared to the Model group, the reductions were 18.86%, 29.96%, and 33.03%, respectively, indicating a decrease in the degree of cell damage. Cell morphology changes are shown in Figure 4E. The Control group exhibited good cell growth with spindle-shaped cells. In the Model group, the number of cells significantly decreased, accompanied by pronounced morphological changes such as cell shrinkage, rounding, and detachment. The cell status in different concentrations of HLTP groups was better than that in the model group. The phenomena of cell shrinkage, rounding, and detachment were reduced, indicating that HLTP can alleviate PC12 cell damage induced by Cort.

The effects of HLTP on the levels of intracellular oxidases and antioxidants in PC12 cells after Cort-induced damage were analyzed. The results are shown in Figures 4F–J. After Cort induction, the activities of SOD and CAT in PC12 cells were significantly reduced (*P* < 0.01), decreasing by 61.51% and 60.23%, respectively, compared to the Control group. This indicates that Cort induction can decrease the activity of intracellular antioxidants. However, after treatment with different concentrations of HLTP (50, 100, and 200 μg/mL), both SOD and CAT activities significantly increased (*P* < 0.01). Specifically, SOD activities increased by 14.77%, 31.36%, and 39.65%, and CAT activities increased by 23.45%, 25.27%, and 35.45%, respectively. Compared to the Control group, after Cort induction, the levels of MDA in PC12 cells significantly increased (*P* < 0.01). After treatment with different concentrations of HLTP (50, 100, and 200 μg/mL), the MDA levels significantly decreased (*P* < 0.05 or 0.01), showing reductions of 16.34%, 27.09%, and 54.66% compared to the Model group. The ROS levels followed a similar trend as MDA and after treatment with different concentrations of HLTP (50, 100, and 200 μg/mL), the ROS levels significantly decreased (*P* < 0.01), showing reductions of 80.78%, 116.9%, and 140.2% compared to the Model group, and exhibited a concentration-dependent trend. The same phenomenon can be observed in the detection of ROS by fluorescence spectrophotometry. It is suggested that HLTP can increase intracellular antioxidant enzyme activity, reduce intracellular reactive oxygen species, alleviate intracellular lipid peroxidation, effectively improve the oxidative stress state in PC12 cells, and achieve a protective effect on nerve cells.

#### 3.3.2 Effects of HLTP on cell apoptosis

**FIGURE 5:**
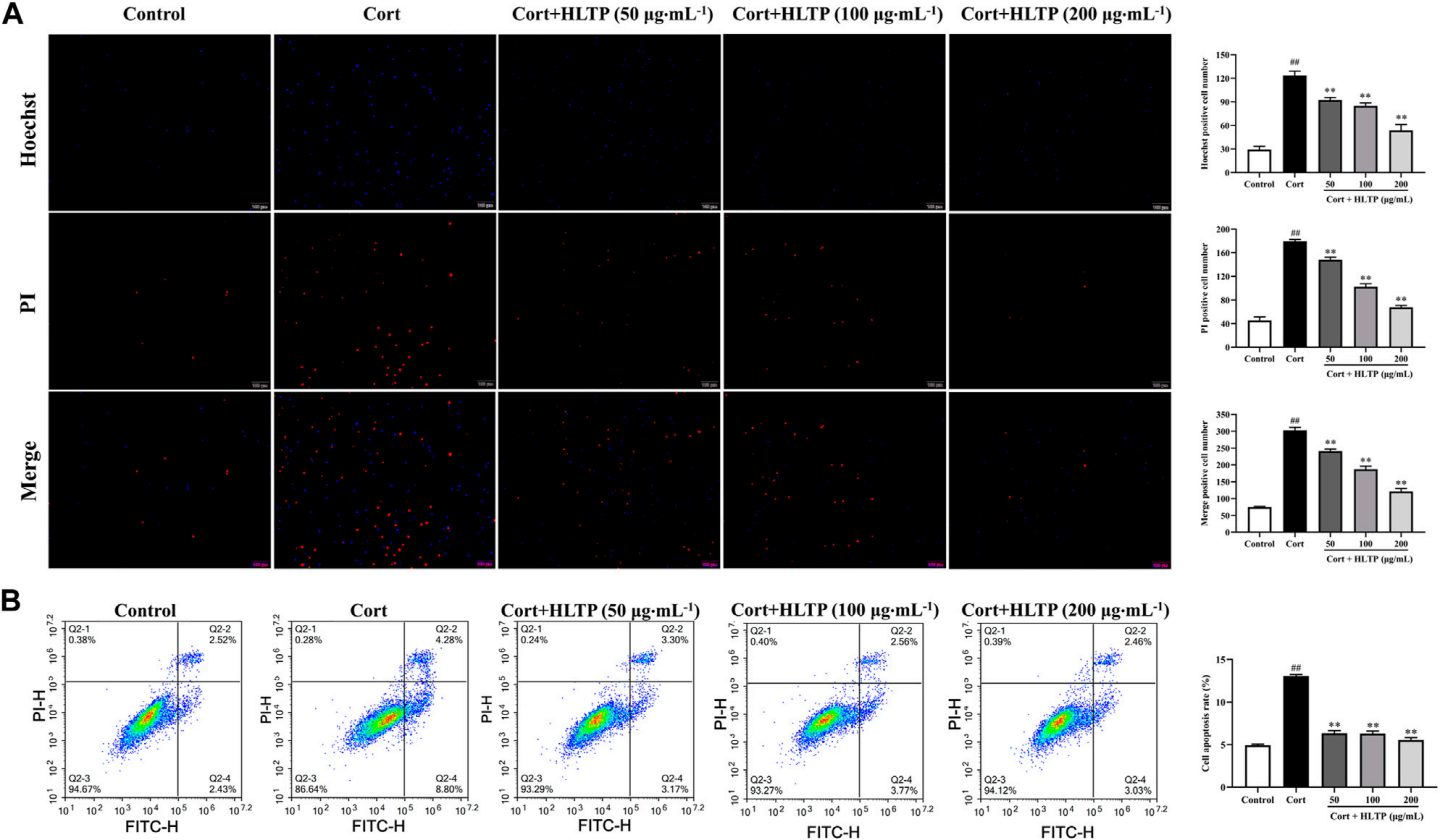
Effects of HLTP on cell apoptosis (**(A)** Hoechst 33342/PI staining to detect cell apoptosis, Hoechst (*F* = 159.6, *P* < 0.0001), PI (*F* = 458.2, *P* < 0.0001), Merge (*F* = 456.2, *P* < 0.0001); **(B)** Flow cytometry to detect cell apoptosis rate, *F* = 585.2, *P*< 0.0001) (Note: Compared with the Control group: ^#^ *P* < 0.05 and ^##^ *P* < 0.01; Compared with the Model group: *P < 0.05 and **P < 0.01; mean ± SD, *n* = 3; **(A, B)** DFn = 4, DFd = 10; Magnification ×20. Fluorescence microscopy and flow cytometry were used to detect the cell apoptosis rate).

The impact of HLTP on the degree of cell apoptosis was analyzed, and the results are shown in Figure 5. Hoechst 33324 is a nucleic acid dye that can enter the cell membrane of normal cells to stain them light blue. Apoptotic cells, with increased membrane permeability, exhibit bright blue fluorescence in their cell nuclei when stained with Hoechst 33324. PI dye cannot penetrate the intact cell membranes of normal cells and apoptotic cells, so live cells resist PI staining, while dead cells are stained red. The Hoechst 33324/PI staining results are shown in Figure 5A. Compared with the Control group, the number of cells emitting bright blue and red fluorescence in the Model group significantly increased (*P* < 0.01), indicating that Cort can cause damage to PC12 cells and induce cell apoptosis, even cell necrosis. In the HLTP group (50, 100, and 200 μg/mL), the number of cells emitting bright blue and red fluorescence significantly decreased (*P* < 0.01), indicating that HLTP can inhibit Cort-induced apoptosis of PC12 cells. After staining PC12 cells with Annexin V-FITC/PI double staining, flow cytometry was used to sort cells in different stages, and the results are shown in Figure 5B. Compared with the Control group, the Q2-2 quadrant (late-stage apoptosis) and Q2-4 quadrant (early-stage apoptosis) cells in the Model group significantly increased, and the cell apoptosis rate significantly increased (*P* < 0.01), indicating that Cort induces apoptosis of PC12 cells. Compared with the Model group, in the HLTP group (50, 100, and 200 μg/mL), the cells in Q2-2 and Q2-4 quadrants decreased, and the cell apoptosis rate decreased. Each concentration of HLTP showed significant differences (*P* < 0.01), indicating that HLTP can exert neuroprotective effects by inhibiting apoptosis of PC12 cells.

#### 3.3.3 Effects of HLTP on MMP and Ca^2+^

**FIGURE 6:**
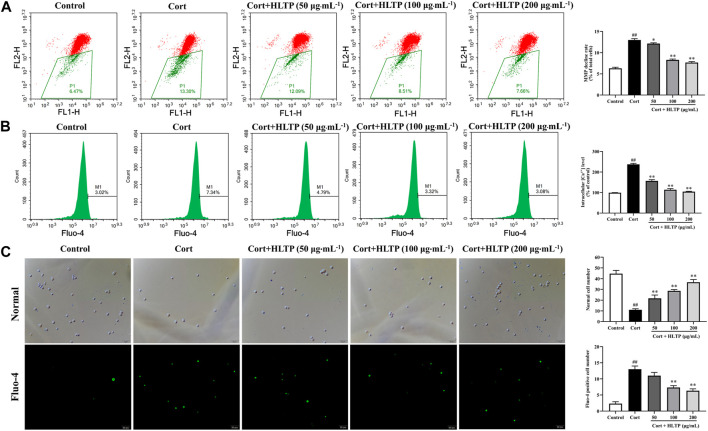
Effects of HLTP on MMP and Ca^2+^ (**(A)** Flow cytometry to detect cellular MMP, *F* = 442.9, *P* < 0.0001; **(B)** Flow cytometry to detect intracellular Ca^2+^ concentration, *F* = 497.1, *P* < 0.0001; **(C)** Fluorescence microscopy to observe intracellular Ca^2+^ concentration, Normal (*F* = 93.4, *P* < 0.0001), Fluo-4 (*F* = 86.7, *P* < 0.0001)) (Note: Compared with the Control group: ^#^ *P* < 0.05 and ^##^ *P* < 0.01; Compared with the Model group: *P < 0.05 and **P < 0.01; mean ± SD, *n* = 3; A–C: DFn = 4, DFd = 10; Magnification ×20. Flow cytometry and fluorescence microscopy were used to detect the MMP and Ca^2+^).

The effects of HLTP on intracellular Ca^2+^ concentration and MMP in injured PC12 cells were analyzed, and the results are shown in Figure 6. When the MMP is high, JC-1 dye aggregates in the matrix of mitochondria, forming polymers that emit red fluorescence. When the MMP is low, JC-1 cannot aggregate in the matrix of mitochondria, and it exists as a monomer, emitting green fluorescence. As shown in Figure 6A, compared with the Control group, the number of cells in the P1 quadrant with lower membrane potential in the Model group significantly increased (*P* < 0.01), increasing by 48.10% compared to the Control group. This indicates that Cort can decrease the MMP in PC12 cells. Compared with the Model group, in the HLTP group (50, 100, and 200 μg/mL), the number of cells in the P1 quadrant decreased, and each concentration of HLTP showed significant differences (*P* < 0.01or 0.05). This suggests that HLTP can effectively restore the depolarization of MMP induced by Cort in PC12 cells, thereby exerting a neuroprotective effect. The Ca^2+^ fluorescence probe Fluo-4 AM itself has almost no fluorescence. Once it enters the cell and undergoes intracellular esterase hydrolysis, the generated Fluo-4 binds to Ca^2+^ and emits green fluorescence, as shown in Figures 6B, C. After Cort induction, the number of cells with green fluorescence increases, and flow cytometry analysis also indicates a significant increase in Ca^2+^ concentration (*P* < 0.01) of 138.0% compared to the Control group. However, after treatment with different concentrations of HLTP (50, 100, and 200 μg/mL), the number of cells with green fluorescence significantly decreases. Flow cytometry analysis also shows a significant reduction in intracellular Ca^2+^ concentration (*P* < 0.01), decreasing by 31.14%, 47.74%, and 51.34%, respectively, compared to the Model group. This indicates that Cort induction of apoptosis in PC12 cells leads to Ca^2+^ overload, and HLTP can effectively alleviate the Ca^2+^ overload effect, exerting a neuroprotective effect.

#### 3.3.4 Effects of HLTP on apoptotic proteins

**FIGURE 7:**
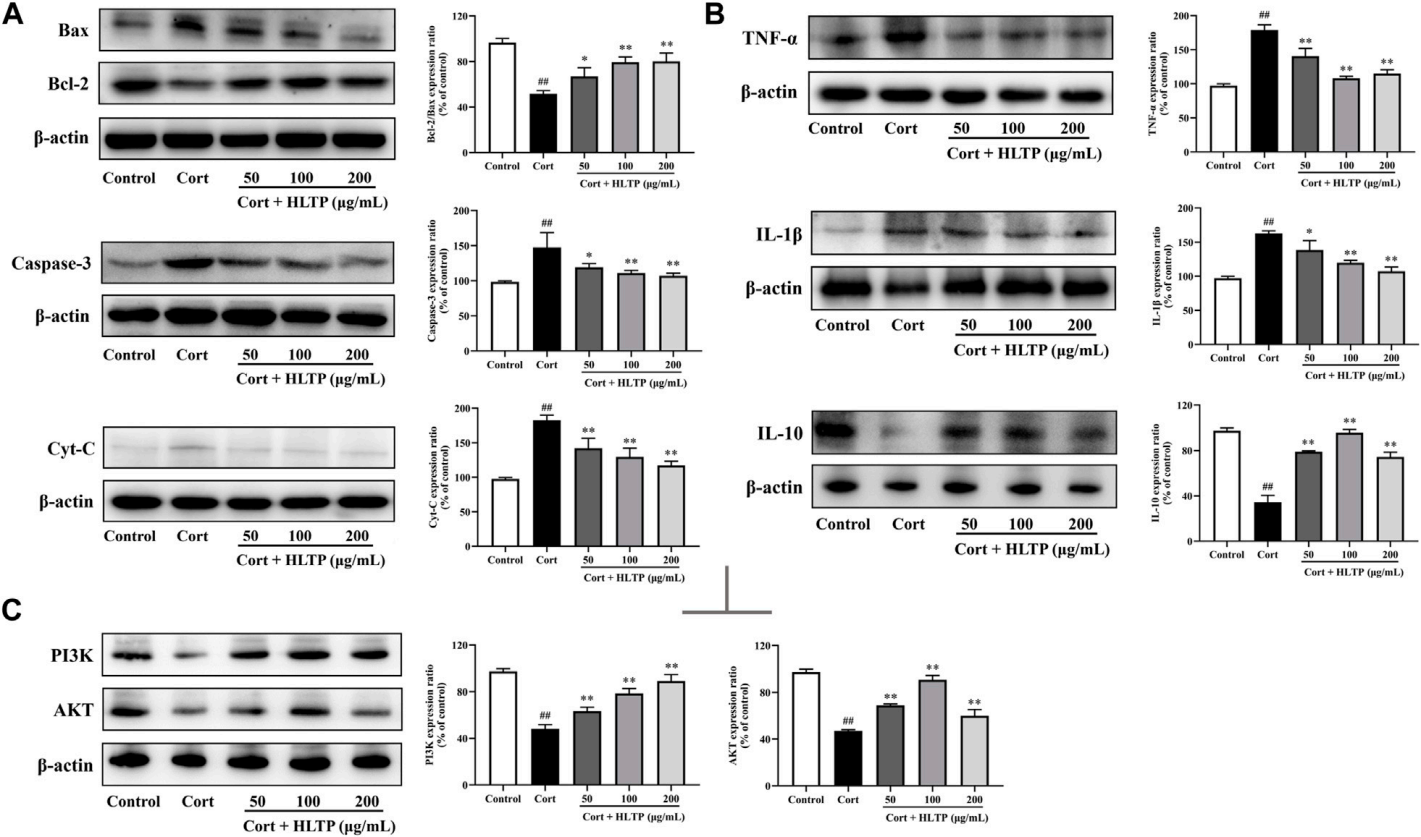
Effects of HLTP on apoptotic proteins, inflammatory factors, and PI3K/AKT signaling pathway (**(A)** Effects of HLTP on apoptotic-related proteins Bcl-2/Bax (*F* = 15.7, *p* = 0.0003), Caspase-3 (*F* = 8.2, *p* = 0.0034), and Cyt-C (*F* = 95.2, *P* < 0.0001); **(B)** Effects of HLTP on cell inflammatory factors TNF-α (*F* = 37.6, *P* < 0.0001), IL-1β (*F* = 22.2, *P* < 0.0001), and IL-10 (*F* = 136.4, *P* < 0.0001); **(C)** Effects of HLTP on the expression of PI3K (F = 76.4, *P* < 0.0001) and AKT (*F* = 128.3, *P* < 0.0001) proteins) (Note: Compared with the Control group: ^#^ *P* < 0.05 and ^##^ *P* < 0.01; Compared with the Model group: *P < 0.05 and **P < 0.01; mean ± SD, *n* = 3; **(A–C)** DFn = 4, DFd = 10. The apoptosis related proteins, inflammatory factors, and PI3K/AKT signaling pathway proteins were detected by Western blotting).

This study examined the expression of apoptosis-related proteins in PC12 cells after treatment with Cort and HLTP. The results, as shown in Figure 7A, indicate that Cort treatment significantly downregulates the expression of the anti-apoptotic protein Bcl-2 and upregulates the expression of pro-apoptotic proteins Bax, Cyt-C, and Caspase-3 (*P* < 0.01). However, treatment with HLTP at concentrations of 50, 100, and 200 μg/mL effectively reverses this effect (*P* < 0.01 or 0.05). The relative expression levels of Bcl-2/Bax proteins increased by 15.27%, 27.90%, and 28.54%, respectively, compared to the Model group. The relative expression levels of Caspase-3 proteins decreased by 28.12%, 36.47%, and 40.32%, respectively, and Cyt-C protein expression decreased by 40.28%, 52.86%, and 65.26%, suggesting that HLTP against Cort-induced apoptosis in PC12 cells by modulating the expression of apoptosis-related proteins.

#### 3.3.5 Effects of HLTP on cellular inflammatory factors

This study examined the expression of inflammatory factors in PC12 cells after treatment with Cort and HLTP. As shown in Figure 7B, Cort treatment significantly increased the protein expression levels of the pro-inflammatory factors TNF-α and IL-1β (*P* < 0.01), while decreasing the anti-inflammatory factor IL-10 (*P* < 0.01). Treatment with different concentrations of HLTP (50, 100, and 200 μg/mL) effectively reversed the abnormal changes in inflammatory factors (*P* < 0.01). Compared to the Model group, the relative protein expression levels of TNF-α decreased by 38.17%, 70.76%, and 63.87%, IL-1β decreased by 24.12%, 42.89%, and 55.38%, and IL-10 increased by 44.61%, 61.32%, and 39.98%. These results suggest that HLTP protect against Cort-induced apoptosis in PC12 cells by modulating the expression of inflammatory factors, thereby alleviating the inflammatory response.

#### 3.3.6 Effects of HLTP on the PI3K/AKT signaling pathway

This study examined the expression of PI3K and AKT proteins in PC12 cells after treatment with Cort and HLTP. As shown in Figure 7C, after 24 h of Cort treatment, the protein expression levels of PI3K and AKT in the cells significantly decreased (*P* < 0.01), with reductions of 49.22% and 50.29% compared to the Control group. However, after treating the cells with different concentrations of HLTP (50, 100, and 200 μg/mL) for 24 h, the protein expression levels of PI3K and AKT in the cells significantly increased (*P*< 0.01). Compared to the Model group, the protein expression levels of PI3K and AKT increased by 15.24%, 30.44%, 41.03%, and 21.78%, 43.63%, 12.86%, respectively. This indicates that HLTP may regulate the PI3K/AKT signaling pathway to inhibit apoptosis in PC12 cells, exerting a neuroprotective effect.

## 4 Discussion

This study employed a combination of network pharmacology and *in vitro* experiments to investigate the neuroprotective effects of HLTP. Initially, based on network topology combined with PPI analysis, we identified that the phenolic components in *H. citrina* leaves exert neuroprotective effects through key targets such as TNF, AKT1, TP53, CASP3, and others. As a core target in the PI3K/AKT pathway, AKT1 plays a crucial role in neuroprotection by contributing to synaptic plasticity and the formation of neurotransmission (Guo et al., 2019; Yang et al., 2012). TNF participates in neuroprotection through various mechanisms, such as mediating 5-HT transport and inhibiting the negative feedback loop of the HPA axis (Schmidt et al., 2019). As a pro-apoptotic factor, TP53 has a crucial role in neuronal apoptosis (Wang et al., 2014). CASP3 can directly cleave poly ADP-ribose polymerase 1 (PARP1), leading to DNA fragmentation and promoting cell apoptosis (Wang et al., 2022). KEGG enrichment analysis revealed that pathways such as serotonin synapse, Ca^2+^ signaling pathway, PI3K/AKT pathway, cell apoptosis pathway, and p53 pathway, which are enriched with numerous targets, may contribute to the neuroprotective effects of *H. citrina* leaves. The 5-HT synaptic pathway consists of various types of 5-HT receptors distributed on presynaptic and postsynaptic membranes in different brain regions. Studies have shown a close correlation between neural damage and 5-HT receptors and their functions (Rincón-Cortés and Grace, 2020). The PI3K/AKT pathway can impact the organism by modulating downstream regulatory proteins through activation or inhibition. This includes the regulation of downstream proteins such as p53 and Bax, which directly or indirectly maintain cell survival (Guo et al., 2019; Ludka et al., 2016). Research has reported that p53 can interact with Bax and Bcl-2 and upregulate Bax expression, downregulate Bcl-2 expression to triggers cell apoptosis and eventually lead to mitochondrial dysfunction (Liao et al., 2014). Due to the neuroprotective effects and mechanisms of phenolic components are mainly focused on flavonoids, but there is evidence suggesting that phenolic acid components also exhibit significant neuroprotective activity (Shi et al., 2019; Wang et al., 2021). Therefore, a total of 32 phenolic components, including both flavonoids and phenolic acids, from *H. citrina* leaves were selected as ligands for molecular docking. The molecular docking results suggest that phenolic acid components and some flavonoid components may be potential active ingredients for neuroprotection in *H. citrina* leaves. The proteins AKT1, PIK3R1, TP53, and PARP1 may be key targets for neuroprotection in *H. citrina* leaves. Among them, PIK3R1 is a major regulatory subtype of PI3K and a key gene in the PI3K/AKT signaling pathway, playing an important role in neuroprotection (Guo et al., 2019; Vallejo-Díaz et al., 2019). PARP1 is an enzyme involved in DNA repair (Sas et al., 2018), and a cleavage substrate of caspase for the apoptotic process, and therefore has an important role in apoptosis (Wenzel et al., 2023). It was further found that the eight core components screened showed neuroprotective effects. In summary, the results from network pharmacology suggest that phenolic components in *H. citrina* leaves may activate the PI3K/AKT pathway to inhibit neuronal damage and exert neuroprotective effects. To validate this hypothesis, a series of *in vitro* experiments were conducted.

Firstly, a Cort-induced PC12 neural cell damage model was established. The results indicate that HLTP have a significant protective effect against Cort-induced neuronal damage. It can significantly reduce the release rate of LDH in cells and decrease the degree of cell damage. Oxidative stress is a biological reaction process triggered by the imbalance between reactive oxygen species (ROS) and antioxidants (SOD, CAT). This imbalance can lead to lipid peroxidation, oxidative damage to proteins and DNA, and ultimately result in cell apoptosis (Liu et al., 2023). Malondialdehyde (MDA) is the product of intracellular lipid peroxidation, and its content reflects the degree of lipid peroxidation and oxidative stress (Mokhtari et al., 2023). Excessive ROS can also damage mitochondria and cell membranes, further leading to cell apoptosis (Zhou et al., 2017). We found that Cort treatment increased ROS and MDA levels in PC12 cells, while reducing SOD and CAT levels. HLTP can significantly reverse these changes, indicating that HLTP can inhibit neuronal damage through antioxidative stress. Mitochondria are the cornerstone of eukaryotic cell life activities, and studies have found abnormal structural and functional states of mitochondria in patients with depression (Yoshimoto et al., 2023). MMP is an important indicator of mitochondrial function. Research have showed that depression can lead to a decrease in MMP, and excessive accumulation of Ca^2+^ can also cause cell apoptosis (Tobe, 2013). Apoptosis involves the expression of various genes and proteins, among which Bax, Caspase-3, and Cyt-C are pro-apoptotic proteins, while Bcl-2 is an anti-apoptotic protein. Bcl-2 can form a heterodimer with Bax, inhibit the release of Cyt-C, further suppress the Caspase cascade reaction, and thus inhibit cell apoptosis (Martin, 2001). In this study, HLTP significantly improved apoptosis, reduced the apoptosis rate, decreased Ca^2+^ levels, increased MMP, and inhibited apoptosis by upregulating the expression of Bcl-2, and downregulating the expression of Bax, Cyt-C, and Caspase-3. This suggests that HLTP can inhibit neuronal damage by improving mitochondrial dysfunction. Research has found that patients with depression have disrupted cellular immune function and abnormal secretion of immune-inflammatory factors (Vollmer et al., 2015). Our results indicate that HLTP can significantly reduce the expression of TNF-α and IL-1β, increase the expression of IL-10 protein, suggesting that the neuroprotective effect of HLTP is related to anti-inflammatory damage.

To elucidate whether the neuroprotective effect of HLTP is mediated through the PI3K/AKT pathway, we conducted Western Blot experiments to validate the key proteins of the PI3K/AKT pathway. The results showed that HLTP significantly increased the expression of PI3K and AKT proteins. Activation of AKT further regulates multiple downstream pathways, including the inhibition of glycogen synthase kinase 3β (GSK3β). GSK3β regulates the most important antioxidant pathway, the Nrf2 pathway, by controlling the subcellular distribution of nuclear factor erythroid 2-related factor 2 (Nrf2) (Rada et al., 2012), thus modulating the imbalance between oxidases and antioxidant enzymes (Chen et al., 2019). Estrogen receptor alpha (ERα) can bind to the regulatory subunit p85α of PI3K, followed by AKT phosphorylation and subsequent ERα phosphorylation. The latter can induce increased transcription of the Bcl-2 gene, ultimately leading to increased expression of the Bcl-2 protein and inhibition of cell apoptosis (Sun et al., 2001). Additionally, AKT also inhibits the downstream transcription factor forkhead box protein O1 (FoxO1), further suppressing the important transcription factor nuclear factor kappa B (NF-κB) that regulates the expression of inflammatory genes, thereby modulating the inflammatory response (Wagatsuma et al., 2016). Therefore, HLTP may regulate the PI3K/AKT pathway, subsequently affecting multiple downstream pathways, leading to the reduction of oxidative damage, mitochondrial dysfunction, and inflammatory injury in PC12 cells, ultimately achieving neuroprotection.

**FIGURE 8:**
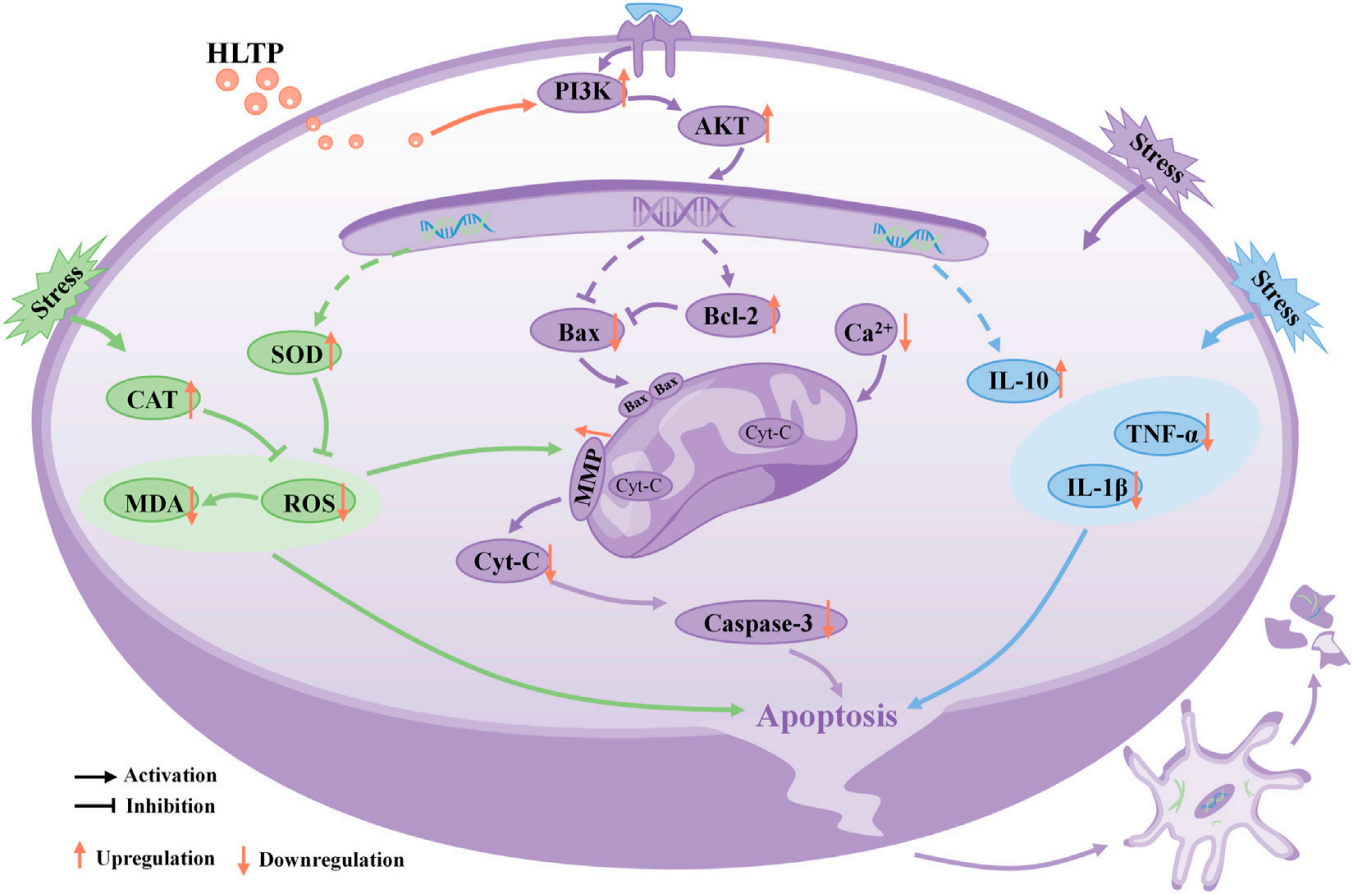
Schematic diagram illustrating the potential molecular mechanisms of neuroprotective effects of HLTP in PC12 cells. (Note: Red upward arrows indicate upregulation of the corresponding target proteins by HLTP, red downward arrows indicate downregulation of the corresponding target proteins by HLTP).

In conclusion, our research results indicate that HLTP enhance the anti-inflammatory, antioxidant, and anti-apoptotic capabilities of PC12 cells, protecting them from Cort-induced damage. The neuroprotective effect is likely mediated through the activation of the PI3K/AKT signaling pathway, as depicted in Figure 8, illustrating the intricate relationship between them.

## 5 Conclusion

In summary, we found that the neuroprotective effect of *H. citrina* leaves is achieved through the synergistic effect of multiple compounds, targets, and pathways and the PI3K/AKT is a crucial pathway for the neuroprotective effects of HLTP. For the first time, we established that HLTP protect damaged neurons through antioxidative stress, amelioration of mitochondrial dysfunction, and mitigation of inflammatory injury. These findings indicate that *H. citrina* leaves could be considered a potential natural resource for treating depression. However, the pathogenesis of depression is complex, and further animal experiments are needed to confirm whether HLTP can also improve animal depressive like behavior by regulating neurotransmitter and gut microbiota disorders.

## Data Availability

The original contributions presented in the study are included in the article/Supplementary material, further inquiries can be directed to the corresponding authors.

## Abbreviations

*H. citrina*, *Hemerocallis citrina* Baroni; HLTP, Hemerocallis citrina Baroni leaves; HPA, Hypothalamic-Pituitary-Adrenal; GCs, Glucocorticoids; LDH, Lactate dehydrogenase; MTT, Thiazolyl Blue Tetrazolium Bromide; DMSO, dimethyl sulfoxide; Cort, Corticosterone; PI3K, Phosphatidylinositol-3-hydroxykinase; AKT, Serine/threonine kinase; SOD, Superoxide dismutase; CAT, Catalase; ROS, Reactive oxygen species; MDA, Malondialdehyde; MMP, Mitochondrial membrane potential; Bcl-2, B cell lymphoma-2; Bax, Bcl-2 Associated X Protein; Cyt-C, Cytochrome C; Caspase-3, Cysteine protease-3; TNF-α, Tumor necrosis factor-α; IL-1β, Interleukin-1β; IL-10, Interleukin-10.
