# Supplementary material for: Neuroprotective effects of total phenolics from Hemerocallis citrina Baroni leaves through the PI3K/AKT pathway
Source: Front Pharmacol. 2024 Jul 12;15:1370619. doi: 10.3389/fphar.2024.1370619 (PMC11272554; doi:10.3389/fphar.2024.1370619)
Supplement: Supplementary file 1 [file Table1.docx]

**Table S1.** Identification components of HLTP with UPLC-Q-TOF-MS data.

| No. | t_R_/min | [M-H]^-^  （m/z） | Adduct | MS^2^（m/z） | Molecular formula | Possible identification | Error (ppm) |
| --- | --- | --- | --- | --- | --- | --- | --- |
| 1 | 5.239 | 353.0889 | -H | 191,179,173,135 | C_16_H_18_O_9_ | Chlorogenic acid | 3.09 |
| 2 | 5.489 | 353.0889 | -H | 191,179,173,135 | C_16_H_18_O_9_ | Neochlorogenic acid | 3.09 |
| 3 | 8.513 | 337.0941 | -H | 191,173,163 | C_16_H_18_O_8_ | 3-p-Coumaroylquinic acid | 3.59 |
| 4 | 9.822 | 353.0887 | -H | 191,179,173,135 | C_16_H_18_O_9_ | 1-O-Caffeoylquinic acid | 2.52 |
| 1* | 10.452 | 367.1044 | -H | 193,173,134 | C_17_H_20_O_9_ | 3-O-Feruloylquinic acid | 2.56 |
| 5 | 10.742 | 353.0890 | -H | 191,179,173,135 | C_16_H_18_O_9_ | 4-O-Caffeoylquinic acid | 3.37 |
| 2* | 12.788 | 337.0941 | -H | 191,173,163 | C_16_H_18_O_8_ | 5-p-Coumaroylquinic acid | 3.59 |
| 6 | 13.263 | 337.0942 | -H | 191,173,163 | C_16_H_18_O_8_ | 4-p-Coumaroylquinic acid | 3.89 |
| 7 | 13.969 | 335.0770 | -H | 179,161,135 | C_16_H_16_O_8_ | 5-O-Caffeoylshikimic acid | -0.72 |
| 3* | 14.697 | 367.1043 | -H | 193,173,134 | C_17_H_20_O_9_ | 5-O-Feruloylquinic acid | 2.29 |
| 8 | 15.084 | 593.1520 | -H | 593,473,353,325,297 | C_27_H_30_O_15_ | Vicenin-2 | 1.37 |
| 4* | 15.387 | 335.0782 | -H | 191,179,173,135 | C_16_H_16_O_8_ | 3-O-Caffeoylshikimic acid | 2.87 |
| 5* | 16.663 | 335.0777 | -H | 191,179,173,135 | C_16_H_16_O_8_ | 4-O-Caffeoylshikimic acid | 1.37 |
| 9 | 16.954 | 755.2042 | -H | 301,300,271,255,243 | C_33_H_40_O_20_ | Quercetin-3-O-α-L-rhamnosyl-(1→6)-[α-L-rhamnosyl-(1→2)]-β-D-glucoside | 0.24 |
|  |  |  | +H | 611,465,303,239,129 |  |  |  |
| 10 | 17.330 | 755.2054 | -H | 755,609,447,301  271,255,243 | C_33_H_40_O_20_ | Quercetin-3-O-α-L-rhamnosyl-(1→6)-β-D-D-glucosyl-(1→2)-α-L-rhamnoside | 1.83 |
| 11 | 17.861 | 609.1475 | -H | 301,271,255,179, 151 | C_27_H_30_O_16_ | Quercetin-3-O-α-L-rhamnosyl-(1→6)-β-D-galactoside | 2.28 |
|  |  |  | +Na | 487,331,325 |  |  |  |
| 12 | 18.355 | 609.1472 | -H | 301,271,255,179, 151 | C_27_H_30_O_16_ | Rutin | 1.79 |
|  |  |  | +Na | 487,331,325 |  |  |  |
| 13 | 18.755 | 739.2100 | -H | 285,284,255 | C_33_H_40_O_19_ | Kaempferol-3-O-rhamnoglucosyl-7-O-rhamnoside | 1.22 |
|  |  |  | +Na | 617,471,309 |  |  |  |
| 15 | 19.722 | 769.2207 | -H | 605,357,339,315,314,300,299,287,271 | C_34_H_42_O_20_ | Isorhamnetin-3-O-rutinoside-7-O-rhamnoside | 1.34 |
|  |  |  | +Na | 647,501,339 |  |  |  |
| 16 | 19.937 | 463.0883 | -H | 301,300,271,255  243,179,151 | C_21_H_20_O_12_ | Hyperoside | 0.22 |
|  |  |  | +Na | 325,185 |  |  |  |
| 17 | 20.513 | 463.0893 | -H | 301,300,271,255  243,178,151 | C_21_H_20_O_12_ | Isoquercitrin | 2.38 |
|  |  |  | +Na | 325,185 |  |  |  |
| 6* | 20.706 | 449.1109 | -H | 449,287,269 | C_21_H_22_O_11_ | Dihydrokaempferol-7-O-β-D-glucoside | 4.36 |
| 18 | 21.062 | 623.1629 | -H | 315,300,272,271,255 | C_28_H_32_O_16_ | Isorhamnetin-3-O-β-D-rutinoside | 1.83 |
|  |  |  | +Na | 647,501,339,331,185 |  |  |  |
| 19 | 21.574 | 579.1367 | -H | 579,301,271,255 | C_26_H_28_O_15_ | Quercetin-3-O-α-L-rhamnose-(1→2)-α-L-arabinopyranoside | 2.00 |
|  |  |  | +H | 581,435,417,303,243 |  |  |  |
| 20 | 21.955 | 433.0785 | -H | 301,300,271,257,  229,179,151 | C_20_H_18_O_11_ | Quercetin-3-O-α-L-arabinopyranoside | 2.01 |
|  |  |  | +Na | 457,325,179,155 |  |  |  |
| 21 | 22.279 | 447.0942 | -H | 285,284,255,227,213 | C_21_H_20_O_11_ | Kaempferol-3-O-α-D-glucoside | 2.04 |
| 22 | 23.464 | 447.0942 | -H | 273,257,245,229,179 | C_21_H_20_O_11_ | Quercetin 3-O-α-L-rhamnoside | 2.04 |
| 24 | 24.033 | 593.1520 | -H | 301,300,271,255,  243,179,151 | C_27_H_30_O_15_ | Quercetin-3,7-O-L-Dirhamnoside | 1.37 |
|  |  |  | +Na | 595, 449,303,163 |  |  |  |
| 25 | 24.936 | 593.1519 | -H | 315,314,300,299,271 | C_27_H_30_O_15_ | Isorhamnetin-3-rhamnose-(1→2)-α-L-arabinopyranoside | 1.20 |
|  |  |  | +Na | 617,471,339,301,193 |  |  |  |
| 7* | 25.324 | 607.1670 | -H | 461,299,285,284  283,267,151 | C_28_H_32_O_15_ | Diosmetin-7-O-rutinoside | 0.26 |
| 26 | 28.460 | 301.0365 | -H | 301,273,245  229,179,151 | C_15_H_10_O_7_ | Quercetin | 3.72 |
| 27 | 31.697 | 269.0434 | +H | 271,153,145,119 | C_15_H_10_O_5_ | Apigenin | -7.99 |
