# Supplementary material for: Neuroprotective effects of total phenolics from Hemerocallis citrina Baroni leaves through the PI3K/AKT pathway
Source: Front Pharmacol. 2024 Jul 12;15:1370619. doi: 10.3389/fphar.2024.1370619 (PMC11272554; doi:10.3389/fphar.2024.1370619)
Supplement: Supplementary file 2 [file Table2.docx]

**Table S2.** Topological results of HLTP exerting neuroprotective effects on target networks.

| Chemical components | Degree | Betweenness Centrality | Closeness Centrality |
| --- | --- | --- | --- |
| M1 | 10 | 0.008286419 | 0.404166667 |
| M2 | 2 | 0.003658277 | 0.37890625 |
| M3 | 13 | 0.020635324 | 0.414529915 |
| M4 | 10 | 0.008286419 | 0.404166667 |
| M5 | 16 | 0.072459249 | 0.425438596 |
| M6 | 12 | 0.02335113 | 0.411016949 |
| M7 | 13 | 0.036086332 | 0.414529915 |
| M8 | 10 | 0.014253435 | 0.404166667 |
| M9 | 17 | 0.078190275 | 0.42920354 |
| M10 | 9 | 0.009215154 | 0.400826446 |
| M11 | 3 | 9.69E-04 | 0.381889764 |
| M12 | 13 | 0.022141413 | 0.414529915 |
| M13 | 9 | 0.011664599 | 0.400826446 |
| M14 | 11 | 0.00557037 | 0.407563025 |
| M15 | 11 | 0.00557037 | 0.407563025 |
| M16 | 12 | 0.006408735 | 0.411016949 |
| M17 | 12 | 0.006408735 | 0.411016949 |
| M18 | 13 | 0.009650405 | 0.414529915 |
| M19 | 12 | 0.006408735 | 0.411016949 |
| M20 | 12 | 0.006408735 | 0.411016949 |
| M21 | 12 | 0.006408735 | 0.411016949 |
| M22 | 12 | 0.042426286 | 0.411016949 |
| M23 | 12 | 0.006408735 | 0.411016949 |
| M24 | 8 | 0.002960088 | 0.397540984 |
| M25 | 12 | 0.006408735 | 0.411016949 |
| M26 | 12 | 0.006408735 | 0.411016949 |
| M27 | 12 | 0.006408735 | 0.411016949 |
| M28 | 13 | 0.009650405 | 0.414529915 |
| M29 | 12 | 0.006408735 | 0.411016949 |
| M30 | 13 | 0.020287709 | 0.414529915 |
| M31 | 43 | 0.259979017 | 0.557471264 |
| M32 | 44 | 0.275131529 | 0.563953488 |
