# Supplementary figures and images for: Neuroprotective effects of total phenolics from Hemerocallis citrina Baroni leaves through the PI3K/AKT pathway
Source: Front Pharmacol. 2024 Jul 12;15:1370619. doi: 10.3389/fphar.2024.1370619 (PMC11272554; doi:10.3389/fphar.2024.1370619)

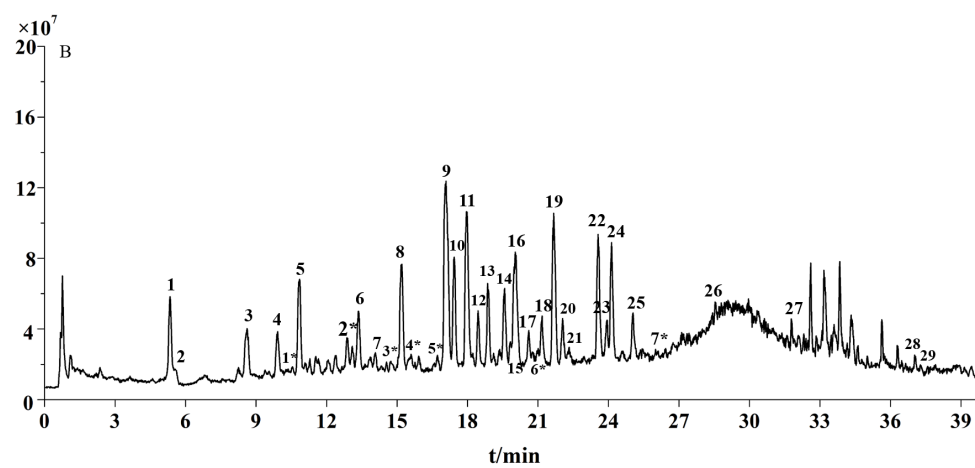

**Figure S1.** TIC(-ESI) of HLTP

Supplement: Supplementary file 3 [file Image1.pdf]
